# Rice *FLOURY SHRUNKEN ENDOSPERM 5* Encodes a Putative Plant Organelle RNA Recognition Protein that Is Required for *cis*-Splicing of Mitochondrial *nad4* Intron 1

**DOI:** 10.1186/s12284-021-00463-2

**Published:** 2021-03-10

**Authors:** Liang Wang, Wenwei Zhang, Shijia Liu, Yunlu Tian, Xi Liu, Haigang Yan, Yue Cai, Xuan Teng, Hui Dong, Rongbo Chen, Xiaokang Jiang, Yihua Wang, Jianmin Wan

**Affiliations:** 1 State Key Laboratory for Crop Genetics and Germplasm Enhancement, Jiangsu Plant Gene Engineering Research Center, Nanjing Agricultural University, Nanjing, 210095 China; 2 National Key Facility for Crop Gene Resources and Genetic Improvement, Institute of Crop Science, Chinese Academy of Agricultural Sciences, Beijing, 100081 China

**Keywords:** *cis*-splicing, Mitochondria, *nad4* intron 1, NADH dehydrogenase, *Oryza sativa*, Seed development

## Abstract

**Background:**

The sequences of several important mitochondrion-encoded genes involved in respiration in higher plants are interrupted by introns. Many nuclear-encoded factors are involved in splicing these introns, but the mechanisms underlying this splicing remain unknown.

**Results:**

We isolated and characterized a rice mutant named *floury shrunken endosperm 5* (*fse5*). In addition to having floury shrunken endosperm, the *fse5* seeds either failed to germinate or produced seedlings which grew slowly and died ultimately. *Fse5* encodes a putative plant organelle RNA recognition (PORR) protein targeted to mitochondria. Mutation of *Fse5* hindered the splicing of the first intron of *nad4*, which encodes an essential subunit of mitochondrial NADH dehydrogenase complex I. The assembly and NADH dehydrogenase activity of complex I were subsequently disrupted by this mutation, and the structure of the mitochondria was abnormal in the *fse5* mutant. The FSE5 protein was shown to interact with mitochondrial intron splicing factor 68 (MISF68), which is also a splicing factor for *nad4* intron 1 identified previously via yeast two-hybrid (Y2H) assays.

**Conclusion:**

*Fse5* which encodes a PORR domain-containing protein, is essential for the splicing of *nad4* intron 1, and loss of *Fse5* function affects seed development and seedling growth.

**Supplementary Information:**

The online version contains supplementary material available at 10.1186/s12284-021-00463-2.

## Background

According to endosymbiotic theory, mitochondria originated from α-proteobacteria ancestors (Gray 1999). Throughout the long-term evolutionary history, most mitochondrial genes have been transferred to the nuclei of their host cells (Timmis et al. 2004). Only a minor portion of mitochondrial genes are retained in angiosperms, and those genes are involved in the electron transport system or encode ribosomal proteins and tRNAs (Kubo and Newton 2008). After these mitochondrial genes are transcribed, their transcripts are processed mainly by nuclear-encoded proteins. This posttranscriptional processing generally includes intron splicing, RNA editing, cleavage, and maturation (Small et al. 2013; Hammani and Giegé 2014).

According to their structural features and splicing mechanisms, mitochondrial introns in flowering plants can be divided into group I members and group II members, with the latter being more predominant (Bonen 2008). Group II introns are involved in both *cis*-splicing and *trans*-splicing. *Cis*-splicing occurs within one pre-mRNA molecule, whereas *trans*-splicing occurs between two pre-mRNA molecules (Sharp 1987; Lasda and Blumenthal 2011).

Various nuclear-encoded proteins are involved in splicing mitochondrial introns in higher plants. Among them, pentatricopeptide repeat (PPR) proteins such as AtMISF26 (Wang et al. 2018), ZmDEK35 (Chen et al. 2017), ZmDEK41/43 (Zhu et al. 2019; Ren et al. 2020), ZmEMP8 (Sun et al. 2018), ZmEMP602 (Ren et al. 2019) and OsFLO10 (Wu et al. 2019) are the most studied splicing factors (Small and Peeters 2000). Other proteins also participate in splicing mitochondrial introns, including the following: the DEXH-box-containing RNA helicases AtABO6 and AtPMH2 (Köhler et al. 2010; He et al. 2012); nuclear-encoded maturases nMAT1 (Nakagawa and Sakurai 2006; Keren et al. 2012), nMAT2 (Keren et al. 2009), and nMAT4 (Cohen et al. 2014); RCC1 family protein RUG3 (Kühn et al. 2011); mitochondrial transcription termination factor mTERF15 (Hsu et al. 2014); and RAD52-like protein ODB1 (Gualberto et al. 2015).

Members of another protein family characterized by their possession of the plant organelle RNA recognition (PORR) domain play an important role in intron splicing in plant organelles. The PORR domain was previously referred to as the “domain of unknown function 860” (DUF860) (http://pfam.xfam.org/family/PF11955) but was later renamed the PORR domain (Kroeger et al. 2009).

To date, several PORR domain-containing proteins have been identified in plants. AtRPD1, a member of the PORR/DUF860 family, has a role in prearranging the maintenance of active cell proliferation during root primordium development. Disruption of the *ROOT PRIMORDIUM DEFECTIVE 1* (*RPD1*) gene causes embryogenesis arrest at the globular to transition stages. In silico structural characterization of RPD1 and RPD1-like proteins implied their possible involvement in various regulatory functions through DNA binding, RNA binding, and protein-protein interactions (Konishi and Sugiyama 2006). *At4g08940*, which encodes a PORR domain-containing protein, is expressed in response to oxidative stress. Compared with wild-type (WT) plants, transgenic *At4g08940-*overexpressing plants were more tolerant to paraquat and cold and less tolerant to t-butyl hydroperoxide and salinity (Luhua et al. 2008). AtWTF9, a mitochondrion-localized PORR domain-containing protein, is required for splicing introns in *ribosomal protein L2* (*rpl2*) and *cytochrome c biogenesis F*_*C*_ (*ccmF*_*C*_). T-DNA insertion alleles *wtf9–1* and *wtf9–2* caused severely stunted shoots and roots; both homozygous mutants were shown to survive to flowering, but the flowers were small and produced only a few milky aborted seeds (Colas des Francs-Small et al. 2012). A recent study showed that mitochondrial HSP60s interact together with WTF9 to regulate intron splicing of *ccmF*_*C*_ and *rpl2* (Hsu et al. 2019).

ZmWTF1, a chloroplast-targeted PORR domain-containing protein, is required for splicing chloroplast-encoded introns. *wtf1–1*, a relatively weak mutant, exhibited a pale green phenotype, whereas mutants *wtf1–3* and *wtf1–4* with null alleles were albino (Kroeger et al. 2009). ZmEMP6, a PORR domain-containing protein located in the mitochondria, is required for both endosperm and embryo development, but the target intron is unclear (Chettoor et al. 2015). To date, no PORR domain-containing protein has been identified in rice.

Here, we report the isolation of *Fse5* in rice though map-based cloning. The *Fse5* allele encodes a mitochondrion-targeted PORR domain-containing protein that is expressed constitutively in various tissues. *Cis*-splicing of *nad4* intron 1 is abolished in the *fse5* mutant, and mitochondrial function and structure are consequently disrupted. Like in other mutants whose mutations cause defective splicing of mitochondrial intron(s), *fse5* seed development and seedling growth were affected. Mature *fse5* seeds exhibited a floury, shrunken phenotype and either failed to germinate or produced weak seedlings that died within 1 month. Taken together, these results indicated that *Fse5* plays an essential role in seed development and subsequent seedling growth.

## Results

### *fse5* Seeds Have a Floury, Shrunken Phenotype

**Fig. 1:**
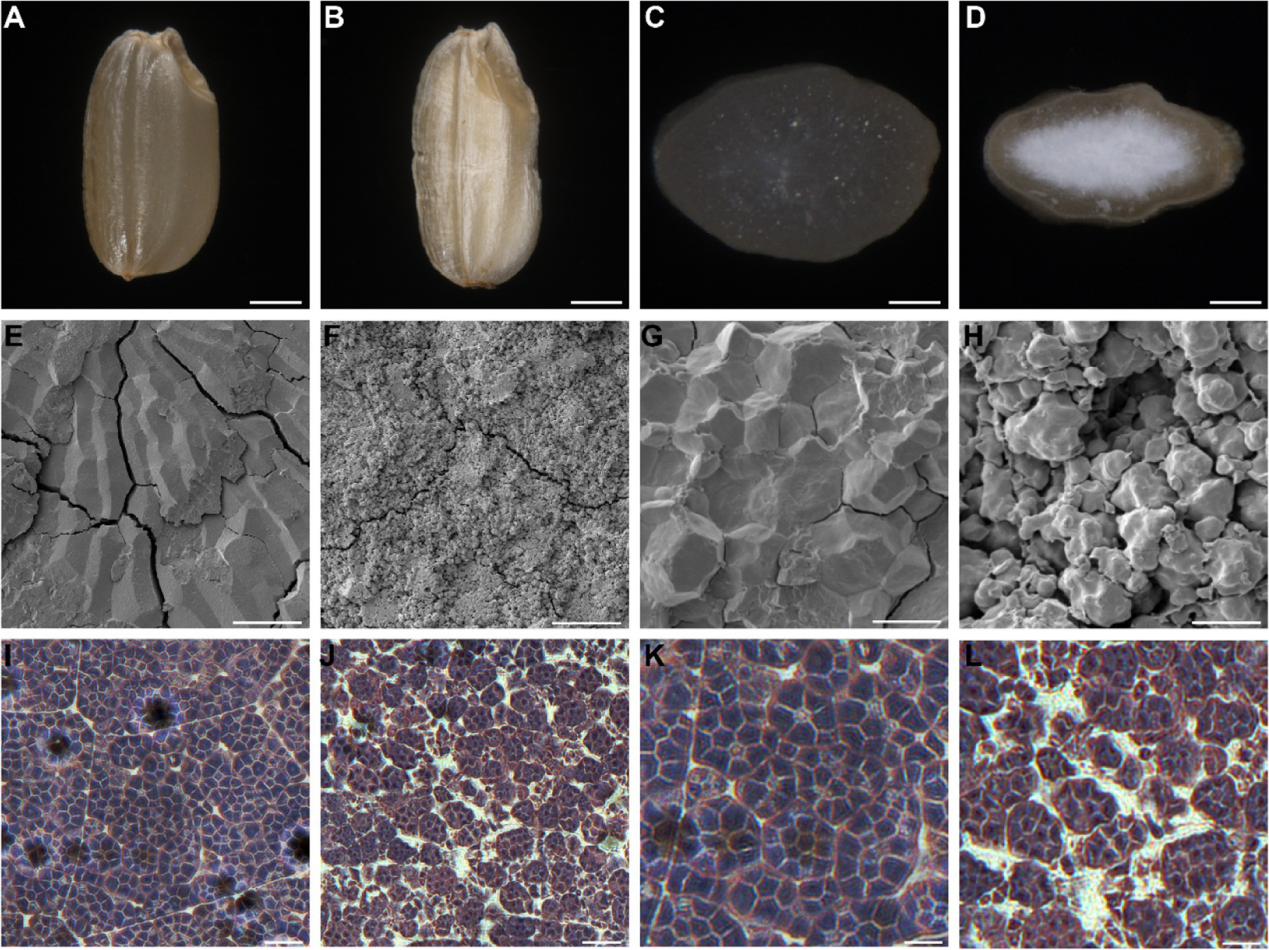
Phenotypes of mature and developing seeds of the WT and *fse5* mutant. **a** and **b** Mature WT (**a**) and *fse5* (**b**) seeds. Bars, 1 mm. **c** and **d** Transverse sections of mature WT (**c**) and *fse5* (**d**) seeds. Bars, 0.5 mm. **e-h** Scanning electron microscopy of transverse sections of mature WT (**e**, **g**) and *fse5* (**f**, **h**) seeds. Bars, 100 μm in (**e**) and (**f**); 10 μm in (**g**) and (**h**). **i**-**l** Iodine-stained semithin endosperm sections of wt (**i**, **k**) and *fse5* (**j**, **l**) seeds at 12 DAF. Bars, 15 μm in (**i**) and (**j**); 7.5 μm in (**k**) and (**l**)

An N-methyl-N-nitrosourea (MNU)-treated population of WT *japonica* cultivar (cv) W017 was generated to study rice endosperm development. A mutant with floury, shrunken endosperm was selected and named *floury shrunken endosperm 5* (*fse5*) (Fig. 1a-d). In addition to the abnormal phenotype, the seeds of the *fse5* mutants failed to germinate or produced seedlings which grew slowly and died within 1 month. Homozygous *fse5* seeds are presumably reproduced by heterozygous plants (+/*fse5*) whose progenies have approximately one-quarter seeds with a mutant phenotype (Additional file 1: Figure S1 and Table S1). Compared with those of the WT W017 seeds, both the 1000-grain weight and grain thickness of mature *fse5* seeds were significantly reduced, but there was no difference in grain length or grain width (Additional file 1: Figure S2A-S2D). In the *fse5* mutant, seed starch and amylose contents were lower (Additional file 1: Figure S2E-S2F), whereas the protein and lipid contents were higher than in the WT (Additional file 1: Figure S2G-S2H). These data showed that the synthesis of seed storage products, especially starch, was significantly affected in *fse5* seeds.

To evaluate defects at the cellular level, observations were made on mature and immature endosperm cells of the WT and *fse5* seeds. Scanning electron microscopy showed that starch grains of mature *fse5* seeds were smooth and loosely packed, whereas those of WT seeds had sharp edges and were tightly assembled (Fig. 1e-h). Compared with those in the WT seeds, starch grains in the *fse5* seeds were irregular and loosely assembled in the developing endosperm cells (Fig. 1i-l). Taken together, these data indicated that the *fse5* mutation affected starch grain development.

### The *fse5* Mutation Causes Embryo and Seedling Death

**Fig. 2:**
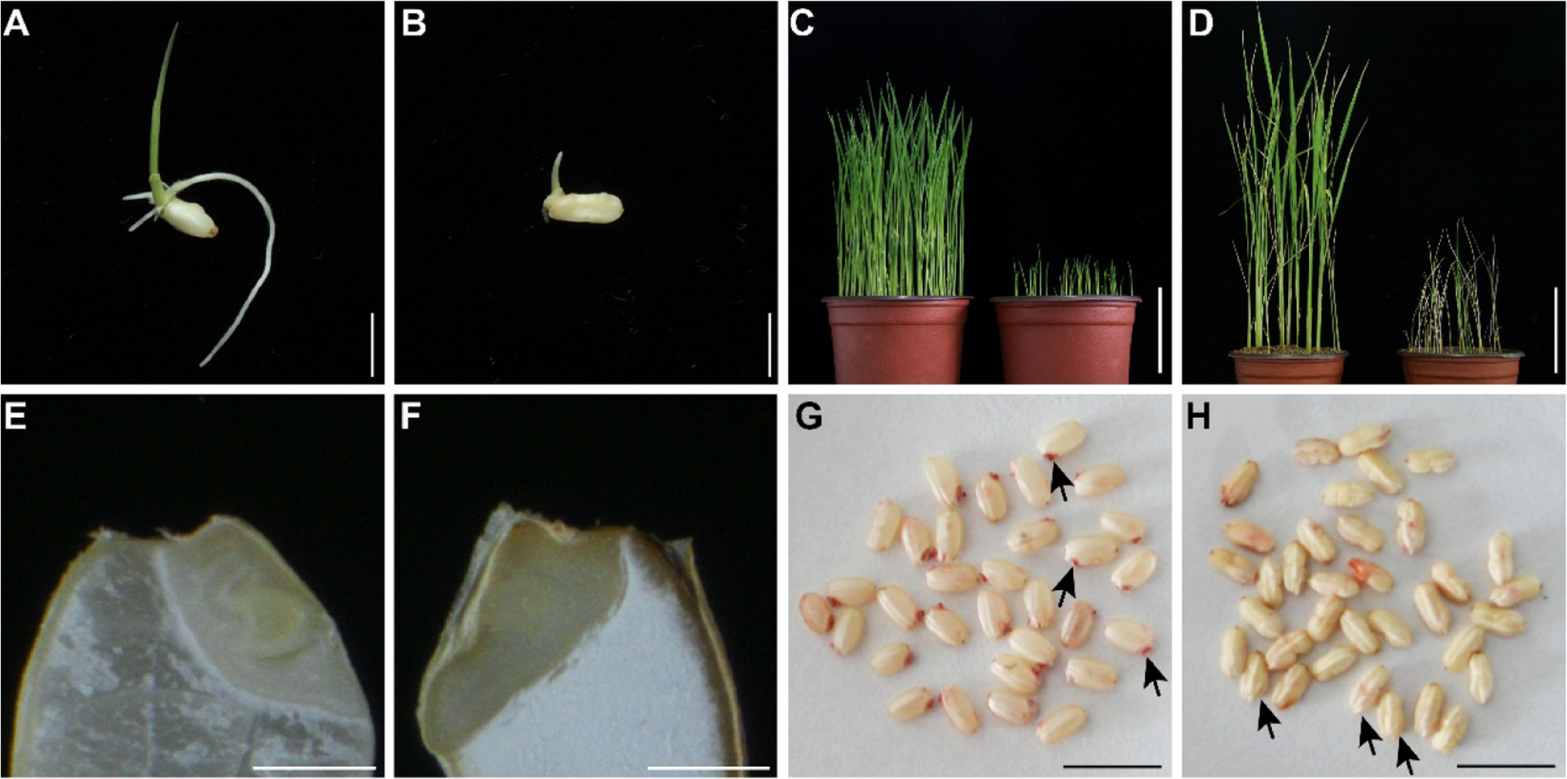
Seed germination and seedling phenotypes. A and B, Germinating seedlings of WT (**a**) and *fse5* mutants (**b**) at 3 DAS. Bars, 5 mm. **c** and **d** Seedlings grown from WT (left) and *fse5* (right) seeds at 9 DAS (**c**) and 30 DAS (**d**). Bars, 5 cm in (**c**) and 10 cm in (**d**). **e** and **f** Longitudinal sections of mature WT (**e**) and *fse5* (**f**) seeds after 24 h of imbibition. Bars, 1 mm. **g** and **h** TTC staining of WT (**g**) and *fse5* (**h**) seeds. The black arrows indicate embryos after staining. Bars, 1 cm

Detailed examination of the germination of the mutant and WT seeds showed that *fse5* seeds with hulls could not germinate, so dehulled seeds were used in further experiments. Compared with that of the WT seeds, the germination percentage of the *fse5* seeds was significantly lower (Additional file 1: Figure S2I), and the plumules and radicles of the latter grew more slowly (Fig. 2a-b). Compared with 94.0% of the WT seeds, only 35.7% of the mutant seeds had produced seedlings by 9 days after sowing (DAS) in the soil (Additional file 1: Figure S2J). The height of *fse5* seedlings reached only one-fourth of that of the WT seedlings (Fig. 2c, Additional file 1: Figure S2K). Moreover, all the *fse5* seedlings were dead by 30 DAS (Fig. 2d). Taken together, these data showed that the *fse5* mutants exhibited seed- or seedling-lethal phenotypes.

To determine the cause of seed or seedling lethality, the embryo structures were observed. Longitudinal sections of seeds after 24 h of imbibition with water revealed clear differentiation in the WT embryos but little change in the *fse5* mutant embryos (Fig. 2e-f). Triphenyl tetrazolium chloride (TTC) staining indicated strong red staining of the WT embryo region, indicating active dehydrogenase activity (Brown et al. 1987), but comparatively little staining of the same tissues in *fse5* seeds (Fig. 2g-h). These results indicated defective embryo differentiation and low seed vigor in the mutant.

### Positional Cloning of *Fse5*

**Fig. 3:**
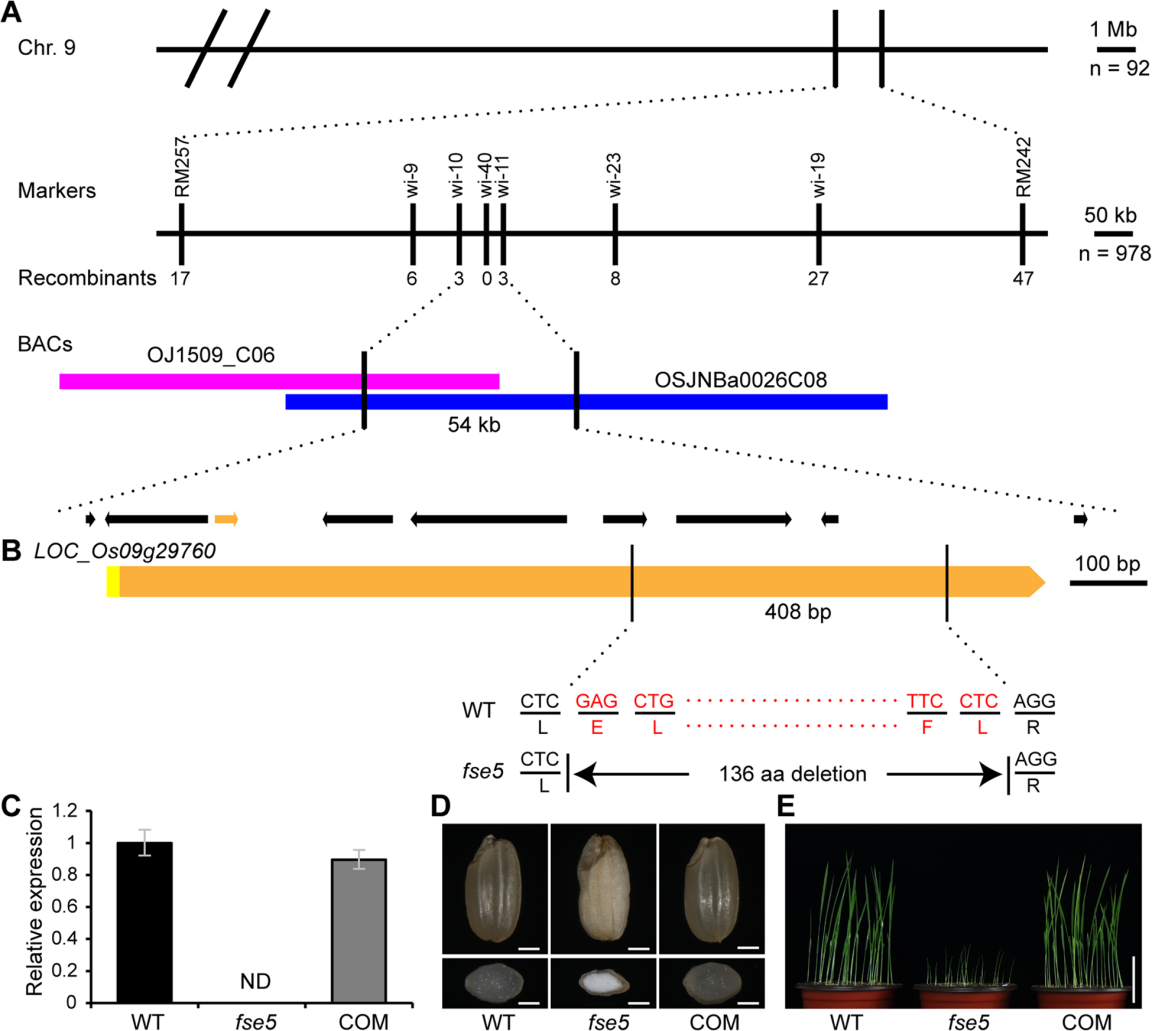
Positional cloning of the *Fse5* locus. **a** The *Fse5* locus was restricted to a 54 kb region flanked by markers wi-10 and wi-11 on chromosome 9 (Chr. 9) and included nine ORFs. Both markers and numbers of recombinants are shown. **b** Mutation site in *LOC_Os09g29760* in the *fse5* mutant. A deleted 408 bp fragment near the end of the exon leads to a 136 aa truncation in the putative encoded protein in the *fse5* mutant. The red letters and dotted lines represent the deletion in *LOC_Os09g29760* or its encoded protein in the *fse5* mutant. The yellow and orange boxes indicate the 5′ untranslated region (UTR) and single exon of *LOC_Os09g29760*, respectively. **c** Expression of *LOC_Os09g29760* in WT, *fse5* mutant and complemented (COM) lines. The primer pair designed within the 408 bp deletion was used in qRT-PCR. Three biological replicates were subjected to qRT-PCR. *OsActin1* was used as an internal control for data normalization. The values are the means ± SDs. ND, not detected. **d** Phenotypes of seeds (upper panel) and transverse sections (lower panel) of WT, *fse5* mutant and COM seeds. Bars, 1 mm. **e** Nine-day-old seedlings of WT, *fse5* mutant and COM lines. Bar, 5 cm

An F_2_ population originating from a cross between a heterozygous (+/*fse5*) plant and Nanjing 11 (*indica*) was used to map the *Fse5* locus. Based on the data from 92 F_2_ individuals, the locus was mapped to a 7.2 cM region flanked by RM242 and RM257 on the long arm of chromosome 9. Nine hundred and seventy-eight F_2_ individuals were used to further delimit the *Fse5* locus to a 54 kb region between insertion/deletion (In/Del) markers wi-10 and wi-11. Nine open reading frames (ORFs) were predicted in this region (Fig. 3a) (http://rice.plantbiology.msu.edu). Sequence analysis revealed a 408 bp deletion in *LOC_Os09g29760* in the *fse5* mutant compared with the WT. The deletion caused a truncation of 136 amino acids (aa) in the *fse5* mutant transcript (Fig. 3b). Thus, *LOC_Os09g29760* was considered to be the candidate gene for *Fse5*.

To further confirm these results, a vector containing the native promoter and WT coding DNA sequence (CDS) of *LOC_Os09g29760* was constructed and introduced into the *fse5* mutant. Quantitative RT-PCR (qRT-PCR) showed that the expression level of *LOC_Os09g29760* was rescued in the positive transgenic lines (Fig. 3c). The dehulled seeds of these lines were transparent and plump, similar to those of the WT (Fig. 3d). Moreover, seedlings grown from these seeds exhibited normal phenotypes (Fig. 3e). Further validation was acquired from *LOC_Os09g29760* knockout (KO) lines produced by CRISPR/Cas9 (Additional file 1: Figure S3A-S3B). The dehulled seeds from four independent KO lines segregated for both normal and mutant seeds in a pattern similar to that of the original mutant (Additional file 1: Figure S3C). Therefore, *LOC_Os09g29760* was demonstrated to be the *Fse5* allele.

### *Fse5* Encodes a PORR Domain-Containing Protein

**Fig. 4:**
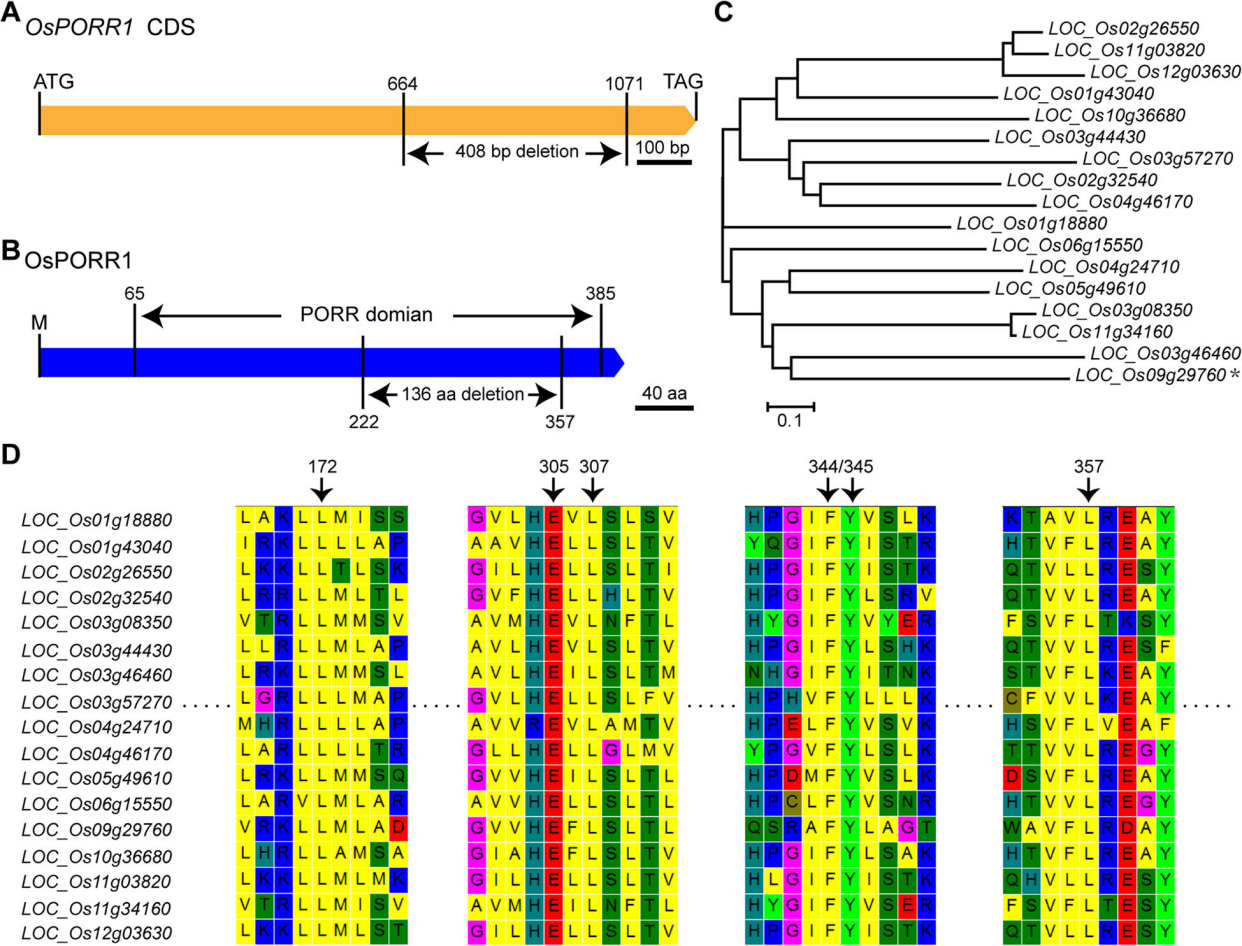
OsPORR1 domain and its phylogenetic analysis. **a** Representation of the deleted 408 bp fragment in the 664–1071 bp region of the *OsPORR1* CDS. **b** The PORR domain is the 65–385 amino acid region of the OsPORR1 sequence. The deleted 136 amino acid fragment is in the region from 222 to 357 aa, within the PORR domain. M, methionine. **c** Phylogenetic analysis of all PORR family members in rice. The black asterisk indicates *OsPORR1*. **d** Multiple sequence alignment of 17 rice PORR domain-containing proteins. The numbers over arrows represent the positions of six highly conserved aa (Leu^172^, Glu^305^, Leu^307^, Phe^344^, Tyr^345^, and Leu^357^)

Sequence analysis showed that the cloned *Fse5* allele contains a single exon (Fig. 4a) and encodes a putative protein of 399 aa (http://rice.plantbiology.msu.edu). The protein was annotated as containing a PORR domain (https://www.ncbi.nlm.nih.gov; https://ricexpro.dna.affrc.go.jp), and the truncation of 136 aa encoded by the 408 bp fragment deleted in the *fse5* allele was within this domain (Fig. 4b). FSE5 was named OsPORR1 because it was the first PORR domain-containing protein identified among the 17 putative PORR domain-containing proteins in rice (Kroeger et al. 2009; Colas des Francs-Small et al. 2012; Chettoor et al. 2015) (Fig. 4c). Multiple sequence alignment showed that 6 aa (Leu^172^, Glu^305^, Leu^307^, Phe^344^, Tyr^345^, and Leu^357^) are highly conserved among all 17 members of the rice family of PORR domain-containing proteins, and all six aa are within the PORR domain, suggesting that they are essential for the function of PORR domain-containing proteins (Fig. 4d). Five of these aa (Leu^172^ is excluded) are present within the truncated 136 aa (Fig. 4b) and therefore likely affect the function of OsPORR1.

The orthologs of *OsPORR1* in maize and Arabidopsis are *GRMZM2G048392* (*ZmEmp6*) and *At4g08940*, respectively (Kroeger et al. 2009; Chettoor et al. 2015). The mutation of *ZmEmp6* causes early arrest in grain development, and compared with WT plants, *At4g08940-*overexpressing transgenic plants are more tolerant to paraquat and cold and less tolerant to t-butyl hydroperoxide and salinity (Luhua et al. 2008; Chettoor et al. 2015). However, the mechanisms underlying these effects remain unknown.

### Spatiotemporal Expression and Subcellular Localization of OsPORR1

**Fig. 5:**
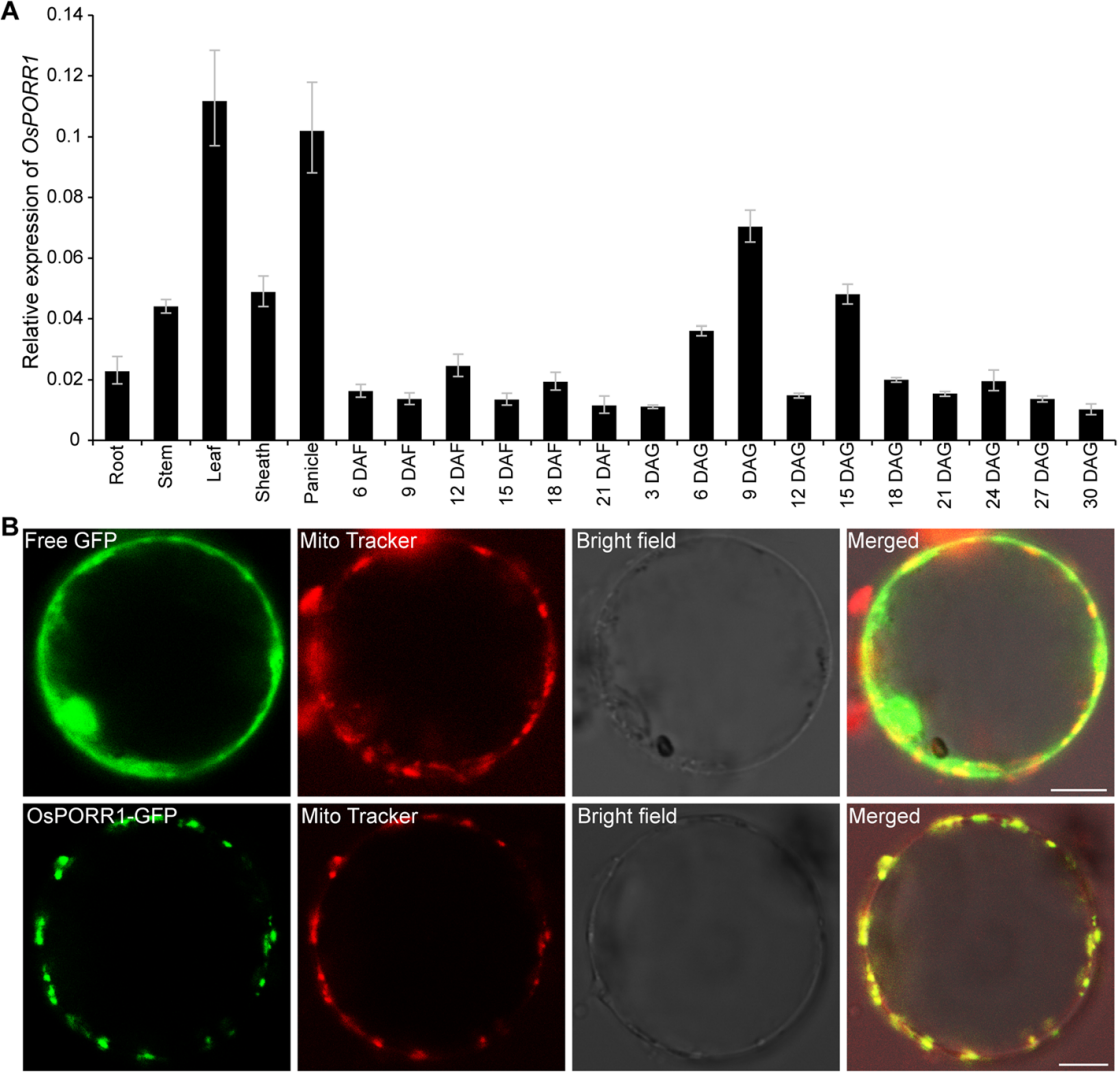
Spatiotemporal expression and subcellular localization of OsPORR1. **a** Expression of *OsPORR1* in various plant organs, developing seeds and seedlings at different times. DAG, days after germination. Three biological replicates were subjected to qRT-PCR. *OsActin1* was used as an internal control for data normalization. The values are the means ± SDs. **b** Subcellular localization of OsPORR1. GFP served as a control. GFP signals in protoplasts isolated from positive transgenic seedlings were observed via confocal laser scanning microscopy. Mito Tracker Orange was used to identify mitochondria. Bars, 10 μm

qRT-PCR analysis showed that *OsPORR1* was expressed in the roots, stems, leaves, leaf sheaths, panicles, developing seeds and seedlings according to the multiple phenotypic characteristics of the *fse5* mutant (Fig. 5a). A vector construct containing the β-glucuronidase (GUS) reporter gene driven by the *OsPORR1* promoter was transformed into rice cv Nipponbare (*japonica*) to confirm the expression of *OsPORR1*; GUS staining was observed in the abovementioned tissues (Additional file 1: Figure S4). Both experiments suggested that *OsPORR1* was constitutively expressed in different tissues and at different growth stages.

OsPORR1 was predicted to localize to the mitochondria based on TargetP analysis (http://www.cbs.dtu.dk/services/TargetP). A vector construct in which a green fluorescent protein (GFP) was fused to the full-length OsPORR1 sequence was transformed into rice cv Nipponbare. The GFP signals in protoplasts isolated from positive transgenic seedlings showed a punctate pattern that overlapped with the orange fluorescence of the mitochondrial indicator Mito Tracker Orange (Fig. 5b). Taken together, these data showed that OsPORR1 was a mitochondrion-targeted protein.

### OsPORR1 Functions in Splicing Mitochondrial *nad4* Intron 1

**Fig. 6:**
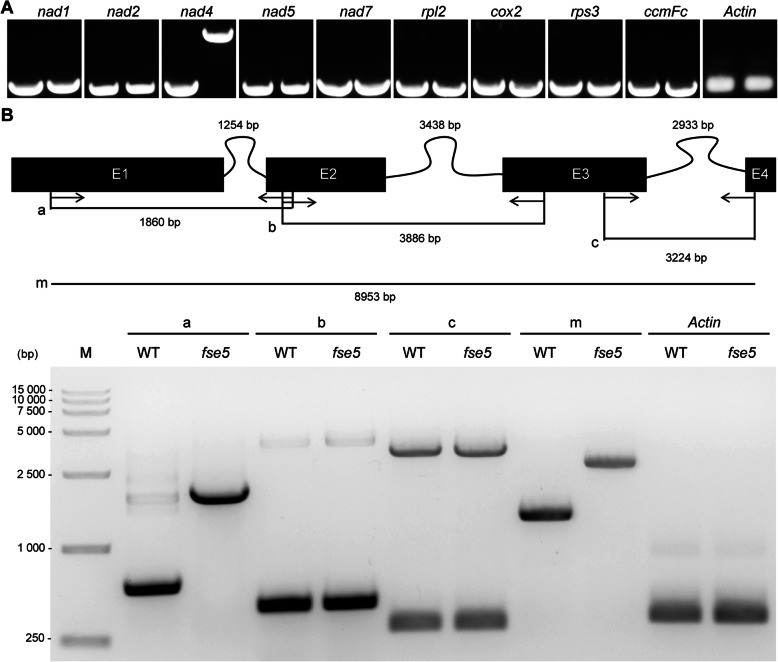
Splicing of *nad4* intron 1 was abolished in the *fse5* mutant. Total RNA was extracted from nine-day-old seedlings, and random hexamer primers were used to synthetize first-strand cDNA. **a**, Transcript analysis of mitochondrial intron-containing genes in the WT (left) and *fse5* mutant (right). *OsActin1* was used as the internal control. Specific primers were used to amplify 9 transcripts (Wu et al. 2019). **b**, Schematic of *nad4* precursor mRNA and RT-PCR analysis of three introns of *nad4*. *nad4* precursor mRNA consists of 4 exons (E1-E4) and 3 introns. The black boxes indicate exons, and the curved lines represent introns. a, b, c, and m are fragments amplified by exon-exon flanking primers (Chen et al. 2017). Numbers of nucleic acids included by the introns and fragments are noted. M, DNA marker

Previous studies have shown that PORR domain-containing proteins usually function in intron splicing of chloroplast- or mitochondrion-encoded genes (Kroeger et al. 2009; Colas des Francs-Small et al. 2012). Based on this premise and their subcellular localization, transcripts of 9 mitochondrion-encoded genes with introns were analyzed*.* The results showed an absence of the mature *nad4* transcript in the *fse5* mutant, but the mutant had a larger *nad4* transcript than the WT did (Fig. 6a). These results suggested that an intron-splicing defect of *nad4* had occurred in the *fse5* mutant. To further confirm the nonspliced intron, each intron of *nad4* was amplified by specific primers in the WT and *fse5* mutant. The results indicated that the splicing of *nad4* intron 1 was abolished and that no mature *nad4* transcript was detected in the *fse5* mutant (Fig. 6b). All mitochondrial introns in rice were examined via qRT-PCR in conjunction with two groups of specific primers. One group was used to amplify the spliced introns, whereas the other group was used for nonspliced introns. Only *nad4* exon 1-exon 2 (spliced fragment) was not detected (ND) in the *fse5* mutant, although its precursor fragment was present (Additional file 1: Figure S5). These data indicated that OsPORR1 specifically affected the splicing of *nad4* intron 1.

### Mutation in OsPORR1 Affects Mitochondrial Structure and Function

**Fig. 7:**
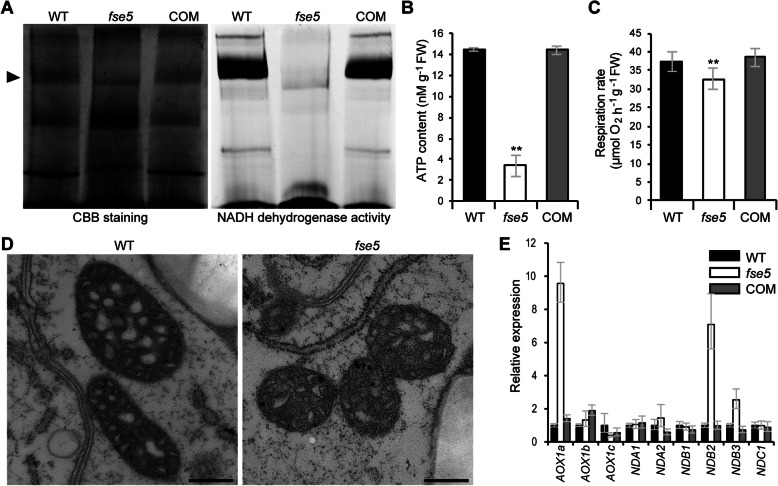
Mitochondrial function and structure were disrupted in *fse5* mutant cells. **a** Mitochondrial complexes separated by BN-PAGE and stained with Coomassie brilliant blue (CBB) (left) and in-gel NADH dehydrogenase activity of complex I (arrow) (right). **b** and **c** Measurement of ATP contents (**b**) and respiration rates (**c**). The values are the means ± SDs. **, *P* < 0.01, Student’s *t* test. **d** Mitochondria in developing endosperm cells of the WT and *fse5* seeds at 9 DAF. Bars, 0.5 μm. **e** Expression of AOX-encoding genes (*AOX1a*, *AOX1b* and *AOX1c*) and alternative NADH dehydrogenase-encoding genes (*NDA1*, *NDA2*, *NDB1*, *NDB2*, *NDB3*, and *NDC1*) in nine-day-old seedlings. Specific primers were used for qRT-PCR (Toda et al. 2012; Li et al. 2013). The data are based on three biological replicates. *OsActin1* was used as an internal control for data normalization. The values are the means ± SDs

Nad4 is an important subunit of complex I in the electron transfer chain of mitochondria. Complex I assembly and activity in seedlings were analyzed by blue native polyacrylamide gel electrophoresis (BN-PAGE). The results showed that the accumulation of complex I in the *fse5* mutant was much lower than that in the WT, and NADH dehydrogenase activity was almost completely abolished (Fig. 7a). The ATP content and respiration rate were also reduced in the mutant compared with the WT (Fig. 7b-c). Complex I is an important component of the mitochondrial inner membrane. Observations were made of the mitochondrial inner structure in developing endosperm cells of the WT and *fse5* mutant. The inner envelope cristae of mitochondria were well organized and surrounded by a dense stroma in the WT, whereas cristae development was poor and the stroma was thin in the *fse5* mutant (Fig. 7d). Previous studies have shown that compromises to the main respiratory chain cause changes in the expression levels of alternative oxidase (AOX)- and NADH dehydrogenase-encoding genes (Toda et al. 2012; Li et al. 2013). Therefore, the transcript levels of AOX-encoding genes (*OsAOX1a*, *OsAOX1b* and *OsAOX1c*) and alternative NADH dehydrogenase-encoding genes (*OsNDA1*, *OsNDA2*, *OsNDB1*, *OsNDB2*, *OsNDB3*, and *OsNDC1*) were measured via by qRT-PCR. The expression of *OsAOX1a*, *OsNDB2* and *OsNDB3* was higher in the *fse5* mutant than in the WT, but there were no differences in expression of the other genes (Fig. 7e). Taken together, these data showed that the defective splicing of *nad4* intron 1 was associated with changes in mitochondrial structure and function.

## Discussion

The *fse5* mutant was isolated via artificial mutagenesis. Like that of other floury endosperm mutants such as *floury endosperm2* (*flo2*) (She et al. 2010), *flo4* (Kang et al. 2005), *flo6* (Peng et al. 2014), *flo7* (Zhang et al. 2016), *flo10* (Wu et al. 2019), *flo11* (Zhu et al. 2018), *flo15* (You et al. 2019), and *flo16* (Teng et al. 2019), the endosperm of *fse5* seeds has loosely packed starch grains. Total starch and amylose contents (Additional file 1: Figure S2E-S2F) and accumulations of major starch synthases granule-bound starch synthase I (GBSSI), starch synthase IIa (SSIIa), starch branching enzyme I (SBEI), and SBEIIb (Additional file 1: Figure S6) were reduced in the *fse5* seeds compared with the WT W017 seeds. Unlike most other floury endosperm mutants, the *fse5* mutant was embryo- or seedling- lethal. OsPORR1 is involved in the splicing of *nad4* intron 1, which is indispensable for normal mitochondrial structure and function. Defects in the splicing of mitochondrion-encoded introns, especially in *NADH dehydrogenase subunit* (*nad*) genes, usually cause aborted seeds, slow-growing seedlings or small plants (Falcon de Longevialle et al. 2007; Koprivova et al. 2010; Liu et al. 2010; Colas des Francs-Small et al. 2014; Yang et al. 2014; Hsieh et al. 2015; Haïli et al. 2016; Xiu et al. 2016; Cai et al. 2017; Chen et al. 2017; Qi et al. 2017; Ren et al. 2017; Dai et al. 2018; Sun et al. 2018; Sun et al. 2019; Wang et al. 2018; Ren et al. 2019; Wu et al. 2019; Zhu et al. 2019; Ren et al. 2020; Yang et al. 2020).

*Emp6*, an ortholog of *OsPORR1* in maize, is also located in mitochondria. Mutant *emp6* kernels show obvious defects in embryos and throughout the endosperm, similar to those of the *fse5* mutant seeds (Chettoor et al. 2015); thus, ZmEMP6 probably functions in grain development in a manner similar to OsFSE5. Multiple sequence alignment showed that the sequences of the PORR domain in ZmWTF1 and AtWTF9 were 49.24% and 38.23% similar, respectively, to that of OsPORR1 (Additional file 1: Figure S7). Both ZmWTF1 and its PORR domain have RNA-binding activity, and ZmWTF1 promotes the splicing of multiple group II introns in chloroplasts (Kroeger et al. 2009). AtWTF9 can also bind RNA and is required for splicing introns of *rpl2* and *ccmF*_*C*_ in mitochondria (Colas des Francs-Small et al. 2012). OsPORR1 is probably involved in the splicing of *nad4* intron 1 by binding to RNA fragments.

Nad4 is an essential subunit of complex I in the mitochondrial respiratory chain. The splicing of *nad4* intron 1 is required for mitochondrial function. Like OsPORR1, ZmDEK35, a mitochondrion-targeted PPR protein, is especially essential for the splicing of *nad4* intron 1. A mutation in *Dek35* affected mitochondrial complex I assembly and NADH dehydrogenase activity, and the *dek35* mutant displayed defective seed development similar to that of the *fse5* mutant (Chen et al. 2017). Intriguingly, small subcomplexes generally considered to be intermediates of complex I were observed by BN-PAGE of the *dek35* mutant but not the *fse5* mutant (Li et al. 2014). A possible explanation is that different tissues of maize and rice were used for BN-PAGE. Homozygous immature *dek35* kernels were used to extract crude mitochondria for BN-PAGE, whereas homozygous seedlings or calli (−/−) are usually used in the case of rice (Hao et al. 2019; Wu et al. 2019; Zheng et al. 2021). In this study, homozygous *fse5* seedlings were used for BN-PAGE. Homozygous immature *dek35* kernels (−/−) are born on heterozygous maternal tissues (+/−) and can acquire maternal nutrients and energy. In those kernels, the defective splicing of *nad4* intron 1 led to dramatically decreased complex I (Chen et al. 2017). The accumulation of subcomplexes may be a response to decreased complex I under coregulation of maternal tissues and mutational kernels. Without support from heterozygous maternal tissues, the response in homozygous seedlings or calli is perhaps weakened or abolished. Thus, small subcomplexes were not observed in the *fse5* mutant or rice *ppr* mutants such as *fgr1*, *flo10* and *osppr939* (Hao et al. 2019; Wu et al. 2019; Zheng et al. 2021).

In addition to ZmDEK35, other PPR proteins such as AtMISF68 (Wang et al. 2018), ZmEMP8 (Sun et al. 2018), ZmEMP602 (Ren et al. 2019) and ZmDEK43 (Ren et al. 2020), and the DEXH-box RNA helicase AtABO6 (He et al. 2012) also affect the splicing of *nad4* intron 1. Their rice orthologs were identified by phylogenetic analysis and subjected to Y2H assays to verify interactions between them. The results of our Y2H assays showed that OsMISF68 interacted together with OsABO6, OsDEK35, OsDEK43, OsEMP8, OsPORR1 and itself (Additional file 1: Figure S8), suggesting a possible functional coordination among these proteins. Whether and how these factors synchronously regulate the splicing of *nad4* intron 1 have yet to be determined.

## Conclusions

*Fse5* encodes a PORR domain-containing protein involved in the splicing of *nad4* intron 1, which is indispensable for normal mitochondrial structure and function. Abnormal mitochondrial cristae and a reduced respiration rate in the *fse5* mutant led to a lower ATP content compared with that in the WT and ultimately affected seed development and viability. These results provide valuable clues for revealing the roles of PORR domain-containing proteins in rice seed development and seedling growth.

## Materials and Methods

### Plant Materials and Growth Conditions

The *fse5* mutant was isolated from an MNU-mutagenized population of W017, a *japonica* variety whose seeds have excellent taste and texture. An F_2_ population for gene mapping was generated by a cross between a heterozygous plant (*+/fse5*) and *indica* cv Nanjing 11. Seed germination tests and seedling growth were subsequently carried out in a growth chamber (12 h light/12 h darkness at 30 °C). Other materials were grown in a paddy field.

### Microscopy

Transverse sections of mature seeds were observed via a Hitachi S-3400 N scanning electron microscope (http://www.hitachi-hitec.com). Scanning electron microscopy was performed as described previously (Kang et al. 2005). Semithin sections of developing seeds at 12 days after flowering (DAF) were observed via light microscope, and sample fixation and sectioning were performed as described previously (Peng et al. 2014).

### Map-Based Cloning of *OsPORR1*

*OsPORR1* was initially mapped using more than 160 polymorphic simple sequence repeat (SSR) and In/Del markers to genotype a small number of mutant F_2_ individuals produced from the abovementioned cross. Fine mapping was then performed with tightly linked molecular markers designed from nucleotide polymorphisms between the Nipponbare and 93–11 reference genomes. The molecular markers used for fine mapping are listed in Additional file 1: Table S2.

### Genetic Complementation

A vector containing the full-length cDNA sequence of *OsPORR1* driven by its native promoter was constructed (the primers used are listed in Additional file 1: Table S3) and transformed into the *fse5* mutant via *Agrobacterium tumefaciens*. Positive transgenic plants were identified by the hygromycin resistance gene used as the selective marker in the pCUbi1390 vector.

### RNA Extraction, Reverse Transcription PCR (RT-PCR) and qRT-PCR

Total RNA was extracted from various tissues using an RNA Prep Pure Plant kit (Tiangen Co., Beijing; http://www.tiangen.com/en/). First-strand cDNA was synthesized using random hexamer primers for mitochondrion-encoded genes and oligo (dT) for nuclear-encoded genes, and PrimeScript Reverse Transcriptase (TaKaRa; http://www.takara-bio.com) was used for reverse transcription. *OsActin1* (*Os03g0718150*) was used as an internal control (Peng et al. 2014). qRT-PCR was performed and the results were analyzed by an ABI 7500 real-time system. The primers used in RT-PCR for nine mitochondrion-encoded genes with introns and for three introns of *nad4* are listed in Additional file 1: Table S4, and the primers used in qRT-PCR for *OsPORR1*, AOX- and alternative NADH dehydrogenase-encoding genes, introns and corresponding exons of mitochondrion-encoded genes are listed in Additional file 1: Table S5.

### Subcellular Localization

The coding region of *OsPORR1* was cloned and inserted in a pCAMBIA1305-GFP vector (the primers used are listed in Additional file 1: Table S3), after which the vector was introduced into rice cv Nipponbare via *Agrobacterium tumefaciens*. Protoplasts of positive seedlings were isolated as described previously (Chen et al. 2006). GFP fluorescent signals were detected by confocal laser scanning microscopy (Leica SP8), and Mito Tracker Orange CMTMRos (Invitrogen, Shanghai) was used to mark the mitochondria.

### BN-PAGE and Complex I Activity Assays

Crude mitochondria were extracted from ten-day-old seedlings grown at 30 °C in darkness (Wang et al. 2017; Wu et al. 2019). BN-PAGE and complex I activity assays were then performed as described previously (Wittig et al. 2006; Hao et al. 2019; Wu et al. 2019).

### Measurement of ATP Content and Respiration Rate

The ATP content of twelve-day-old seedlings grown at 30 °C in darkness was measured with an ATP assay kit (Beyotime, Shanghai), and the respiration rate of nine-day-old seedlings grown at 30 °C was measured by a liquid-phase oxygen electrode (Hansatech, UK).

## Supplementary Information

**Additional file 1: Figure S1.** Homozygous *fse5* seeds were produced by a heterozygous plant (+/*fse5*). Segregation of grains with normal vitreous and floury (black, red arrows) kernels occurred among all seeds harvested from a single heterozygous plant (+/*fse5*) and was viewed by an X-ray viewer (PD-HA). Bar, 1 cm. **Figure S2.** Phenotypes of seeds and seedlings of the WT and *fse5* mutant lines. A-D, 1000-grain weight (A), and length (B), width (C) and thickness (D) of mature WT and *fse5* seeds. E-H, Total starch (E), amylose (F), protein (G) and lipid (H) contents of mature WT and *fse5* seeds. I, Germination percentages for WT and *fse5* seeds at 7 DAS in culture dishes. J, Percentages of seedlings grown from WT and *fse5* seeds at 9 DAS in soil. K, Heights of 9-day-old seedlings grown from WT and *fse5* seeds. The values are the means ± SDs. **, *P* < 0.01, Student’s *t* test. **Figure S3.** *OsPORR1* KO lines generated via CRISPR/Cas9. A, KO target site in the genomic sequence of *OsPORR1*. B, Target sequences of the *OsPORR1* allele in four independent positive lines of Nipponbare. Single-nucleotide insertions (red letters, A, T and G) occurred in KO-140, KO-155 and KO-172, and a 32-nucleotide deletion (red dotted line) was present in KO-174. The black box indicates the protospacer-adjacent motif (PAM) sequence. C, Phenotypes (upper panel) and transverse sections (lower panel) of seeds from Nipponbare and KO lines. Bars, 1 mm. **Figure S4.** GUS staining of various tissues from a *ProOsPORR1:GUS* transgenic plant. Left-to-right, young seedling, root, stem, leaf, leaf sheath, panicle and developing seed. The promoter of *OsPORR1* was inserted into a pCAMBIA1381Z vector, which was then introduced into Nipponbare via *Agrobacterium tumefaciens* transformation. GUS staining was performed as described previously (Zhang and Muench, 2015). Bars, 2 mm. **Figure S5.** qRT-PCR analysis of 23 mitochondrial introns. Primers spanning adjacent exons were used to amplify fragments of mature mitochondrial transcripts (upper panel), and primers spanning adjacent exons and introns were used to amplify fragments of mitochondrial precursor mRNAs (lower panel) (Cai et al. 2017; Chen et al. 2017; Lee et al., 2017). The results of *nad4* exon 1-exon 2 (spliced fragment) and its precursor fragment are indicated by a black box. ND, not detected. Three biological replicates were assessed via qRT-PCR, and *OsActin1* was used as an internal control for data normalization. The values are the means ± SDs. **Figure S6.** Immunoblot analysis of major starch synthases. Total proteins were extracted from developing WT and *fse5* seeds at 15 DAF and separated by SDS-PAGE (Takemoto et al., 2002; Wang et al., 2010). Translation elongation factor 1α (EF-1α), encoded by *Os03g0177400*, served as the loading control. GBSSI, Granule-bound starch synthase I. SSIIa, Starch synthase IIa. SBE, Starch branching enzyme. AGPS2b, ADP-glucose pyrophosphorylase 2b. AGPL2, ADP-glucose pyrophosphorylase large subunit 2. **Figure S7.** Multiple sequence alignment of PORR domains in OsPORR1, ZmWTF1 and AtWTF9. ZmWTF1, *GRMZM2G403797*; AtWTF9, *At2g39120*. **Figure S8.** Y2H assays showing that MISF68 interacts together with splicing factors of *nad4* intron 1. Empty-AD and Empty-BK were used as controls. Full-length cDNAs of *OsABO6* (*LOC_Os01g02884*), *OsDEK35* (*LOC_Os03g50500*), *OsDEK43* (*LOC_Os05g11700*), *OsMISF68* (*LOC_Os02g16650*), *OsEMP8* (*LOC_Os08g41380*) and *OsPORR1* were cloned into pGADT7 or pGBKT7 vectors. The yeast transformation and screening procedures were performed according to the manufacturer’s instructions (TaKaRa Bio, Kusatsu, Japan). DDO, Double dropout media (SD/−Trp-Leu). QDO, Quadruple dropout media (SD/−Trp-Leu-His-Ade). **Table S1.** Segregation of vitreous and floury grains from seven heterozygous plants (+/*fse5*). **Table S2.** Primers used for mapping. **Table S3.** Primers used for vector construction. **Table S4.** Primers used for splicing analysis. **Table S5.** Primers used for qRT-PCR analysis.

## Data Availability

All data supporting the conclusions of this article are provided within the article (and its additional files).

## Abbreviations

ABO: ABA OVERLY SENSITIVE
AGPL2: ADP-glucose pyrophosphorylase large subunit 2
AGPS2b: ADP-glucose pyrophosphorylase small subunit 2b
AOX: Alternative oxidase
BN-PAGE: Blue native polyacrylamide gel electrophoresis
CBB: Coomassie brilliant blue
*ccmF*_*C*_: *Cytochrome c biogenesis F*_*C*_
CDS: Coding DNA sequence
COM: Complemented
*cox2*: *Cytochrome c oxidase subunit II*
cv: Cultivar
DAF: Days after flowering
DAG: Days after germination
DAS: Days after sowing
DEK: Defective kernel
EF-1α: Translation elongation factor 1α
EMP: Empty pericarp
FLO: Floury endosperm
FSE5: FLOURY SHRUNKEN ENDOSPERM 5
GBSSI: Granule-bound starch synthase I
GFP: Green fluorescent protein
GUS: β-glucuronidase
HSP60: Heat shock protein 60
In/Del: Insertion/Deletion
KO: Knockout
MISF: Mitochondrial intron splicing factor
MNU: N-methyl-N-nitrosourea
*nad*: *NADH dehydrogenase subunit*
ND: Not detected
NDA: Alternative NADH dehydrogenase A
NDB: Alternative NADH dehydrogenase B
NDC: Alternative NADH dehydrogenase C
ORF: Open reading frame
PAM: Protospacer-adjacent motif
PORR: Plant organelle RNA recognition
PPR: Pentatricopeptide repeat
qRT-PCR: Quantitative RT-PCR
RPD1: ROOT PRIMORDIUM DEFECTIVE 1
*rpl2*: *Ribosomal protein L2*
*rps3*: *Ribosomal protein S3*
SBE: Starch branching enzyme
SSIIa: Starch synthase IIa
TTC: Triphenyl tetrazolium chloride
UTR: Untranslated region
WT: Wild type
WTF: What’s this factor
Y2H: Yeast two-hybrid assay
