# Supplementary material for: Rice FLOURY SHRUNKEN ENDOSPERM 5 Encodes a Putative Plant Organelle RNA Recognition Protein that Is Required for cis-Splicing of Mitochondrial nad4 Intron 1
Source: Rice (N Y). 2021 Mar 10;14:29. doi: 10.1186/s12284-021-00463-2 (PMC7947098; doi:10.1186/s12284-021-00463-2)
Supplement: Supplementary file 1 — Additional file 1: Figure S1. Homozygous fse5 seeds were produced by a heterozygous plant (+/fse5). Segregation of grains with normal vitreous and floury (black, red arrows) kernels occurred among all seeds harvested from a single heterozygous plant (+/fse5) and was viewed by an X-ray viewer (PD-HA). Bar, 1 cm. Figure S2. Phenotypes of seeds and seedlings of the WT and fse5 mutant lines. A-D, 1000-grain weight (A), and length (B), width (C) and thickness (D) of mature WT and fse5 seeds. E-H, Total starch (E), amylose (F), protein (G) and lipid (H) contents of mature WT and fse5 seeds. I, Germination percentages for WT and fse5 seeds at 7 DAS in culture dishes. J, Percentages of seedlings grown from WT and fse5 seeds at 9 DAS in soil. K, Heights of 9-day-old seedlings grown from WT and fse5 seeds. The values are the means ± SDs. **, P < 0.01, Student’s t test. Figure S3. OsPORR1 KO lines generated via CRISPR/Cas9. A, KO target site in the genomic sequence of OsPORR1. B, Target sequences of the OsPORR1 allele in four independent positive lines of Nipponbare. Single-nucleotide insertions (red letters, A, T and G) occurred in KO-140, KO-155 and KO-172, and a 32-nucleotide deletion (red dotted line) was present in KO-174. The black box indicates the protospacer-adjacent motif (PAM) sequence. C, Phenotypes (upper panel) and transverse sections (lower panel) of seeds from Nipponbare and KO lines. Bars, 1 mm. Figure S4. GUS staining of various tissues from a ProOsPORR1:GUS transgenic plant. Left-to-right, young seedling, root, stem, leaf, leaf sheath, panicle and developing seed. The promoter of OsPORR1 was inserted into a pCAMBIA1381Z vector, which was then introduced into Nipponbare via Agrobacterium tumefaciens transformation. GUS staining was performed as described previously (Zhang and Muench, 2015). Bars, 2 mm. Figure S5. qRT-PCR analysis of 23 mitochondrial introns. Primers spanning adjacent exons were used to amplify fragments of mature mitochondrial transcripts (up [file 12284_2021_463_MOESM1_ESM.docx]

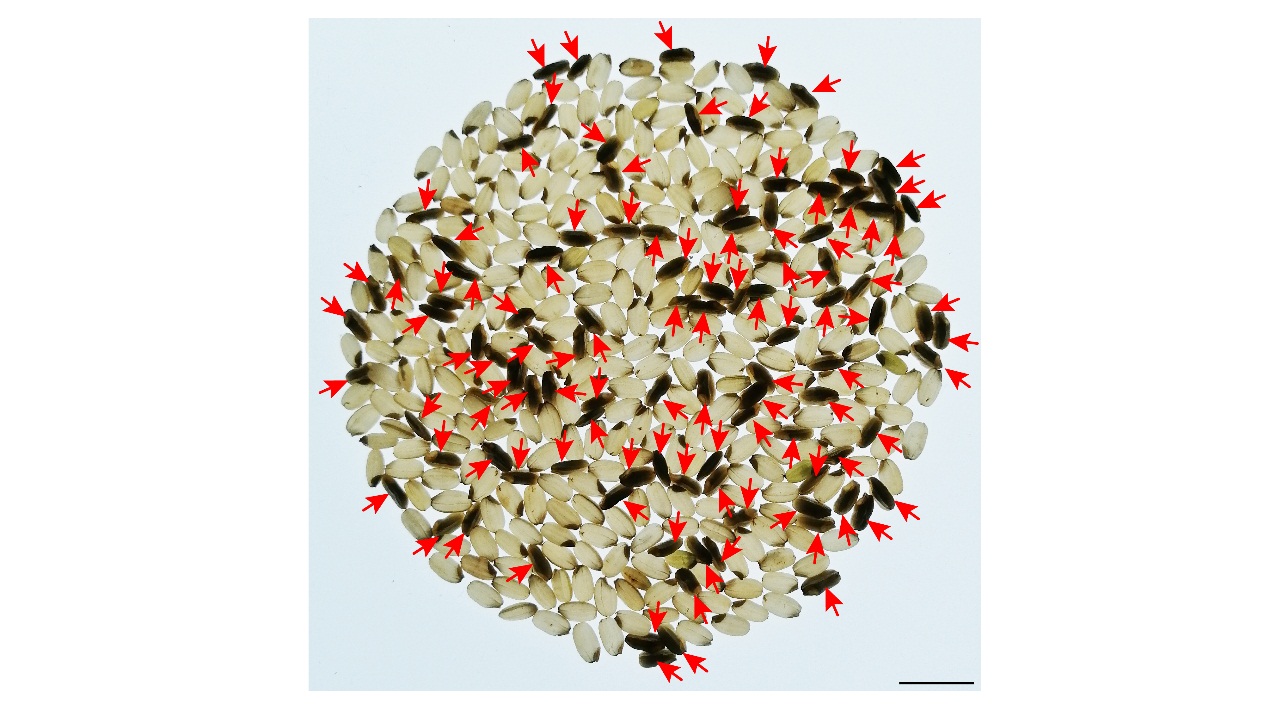


**Figure S1.** Homozygous *fse5* seeds were produced by a heterozygous plant (+/*fse5*). Segregation of grains with normal vitreous and floury (black, red arrows) kernels occurred among all seeds harvested from a single heterozygous plant (+/*fse5*) and was viewed by an X-ray viewer (PD-HA). Bar, 1 cm.


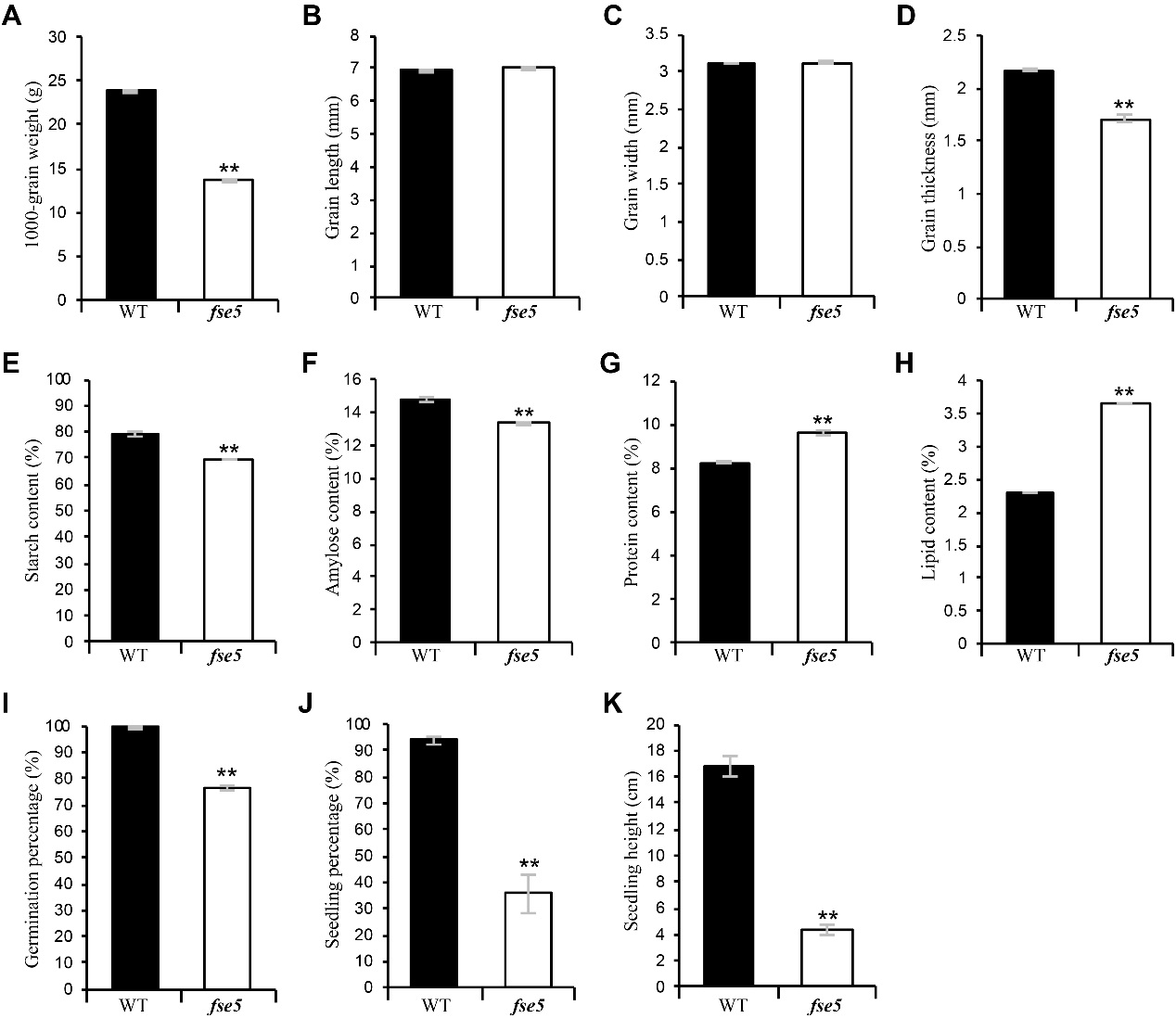


**Figure S2.** Phenotypes of seeds and seedlings of the WT and *fse5* mutant lines. A-D, 1000-grain weight (A), and length (B), width (C) and thickness (D) of mature WT and *fse5* seeds. E-H, Total starch (E), amylose (F), protein (G) and lipid (H) contents of mature WT and *fse5* seeds. I, Germination percentages for WT and *fse5* seeds at 7 DAS in culture dishes. J, Percentages of seedlings grown from WT and *fse5* seeds at 9 DAS in soil. K, Heights of 9-day-old seedlings grown from WT and *fse5* seeds. The values are the means ± SDs. **, *P* < 0.01, Student’s *t* test.

**
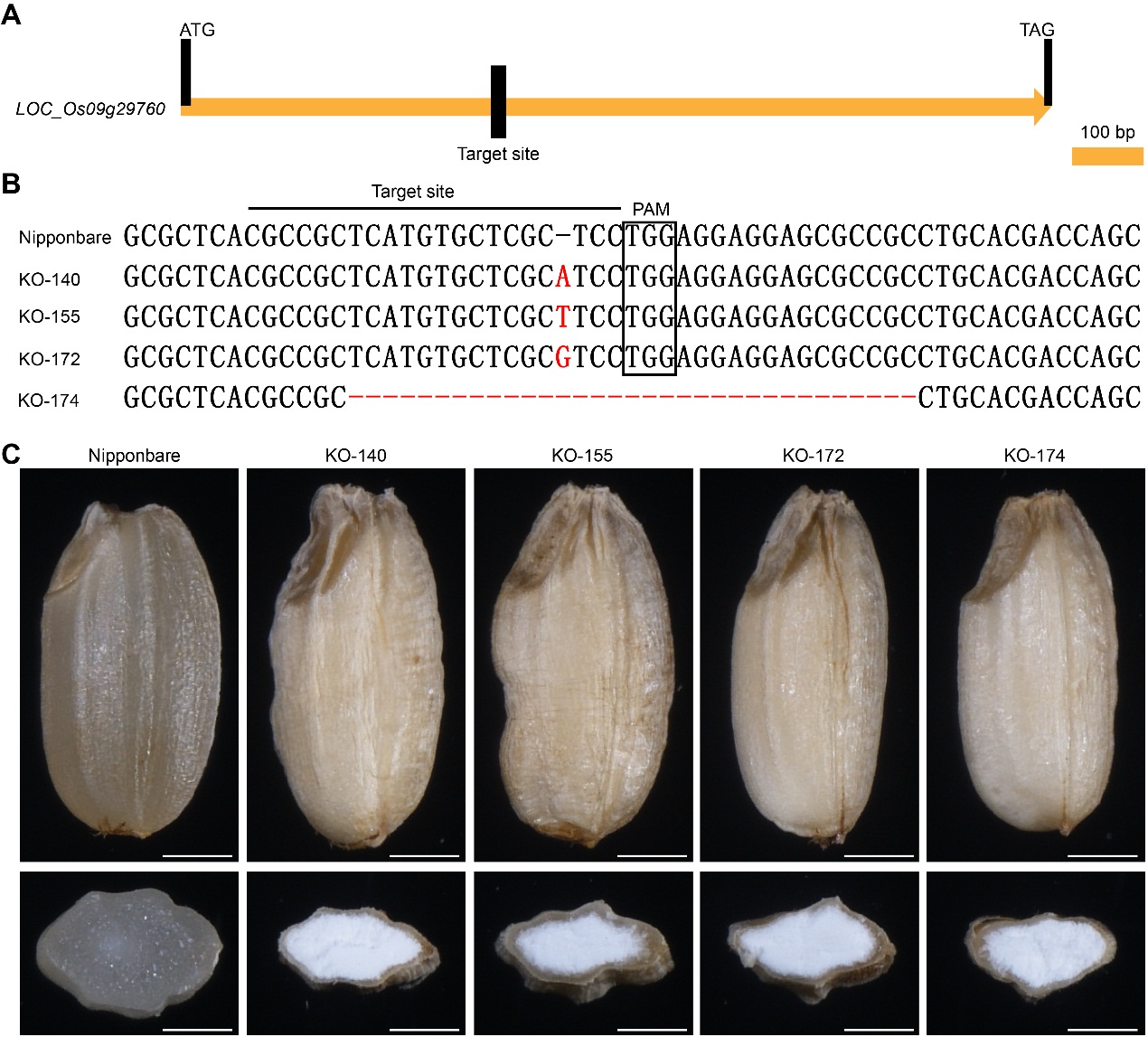
**

**Figure S3.** *OsPORR1* KO lines generated via CRISPR/Cas9. A, KO target site in the genomic sequence of *OsPORR1*. B, Target sequences of the *OsPORR1* allele in four independent positive lines of Nipponbare. Single-nucleotide insertions (red letters, A, T and G) occurred in KO-140, KO-155 and KO-172, and a 32-nucleotide deletion (red dotted line) was present in KO-174. The black box indicates the protospacer-adjacent motif (PAM) sequence. C, Phenotypes (upper panel) and transverse sections (lower panel) of seeds from Nipponbare and KO lines. Bars, 1 mm.


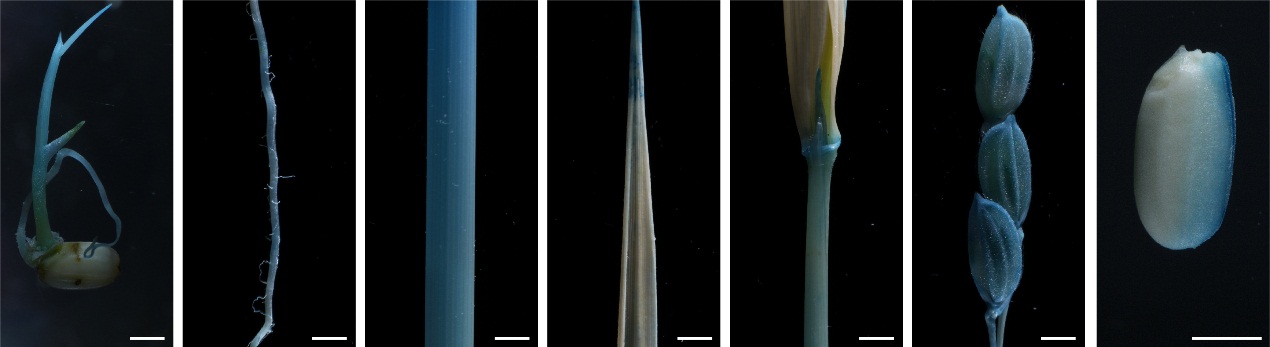


**Figure S4.** GUS staining of various tissues from a *ProOsPORR1:GUS* transgenic plant. Left-to-right, young seedling, root, stem, leaf, leaf sheath, panicle and developing seed. The promoter of *OsPORR1* was inserted into a pCAMBIA1381Z vector, which was then introduced into Nipponbare via *Agrobacterium tumefaciens* transformation. GUS staining was performed as described previously (Zhang and Muench, 2015). Bars, 2 mm.


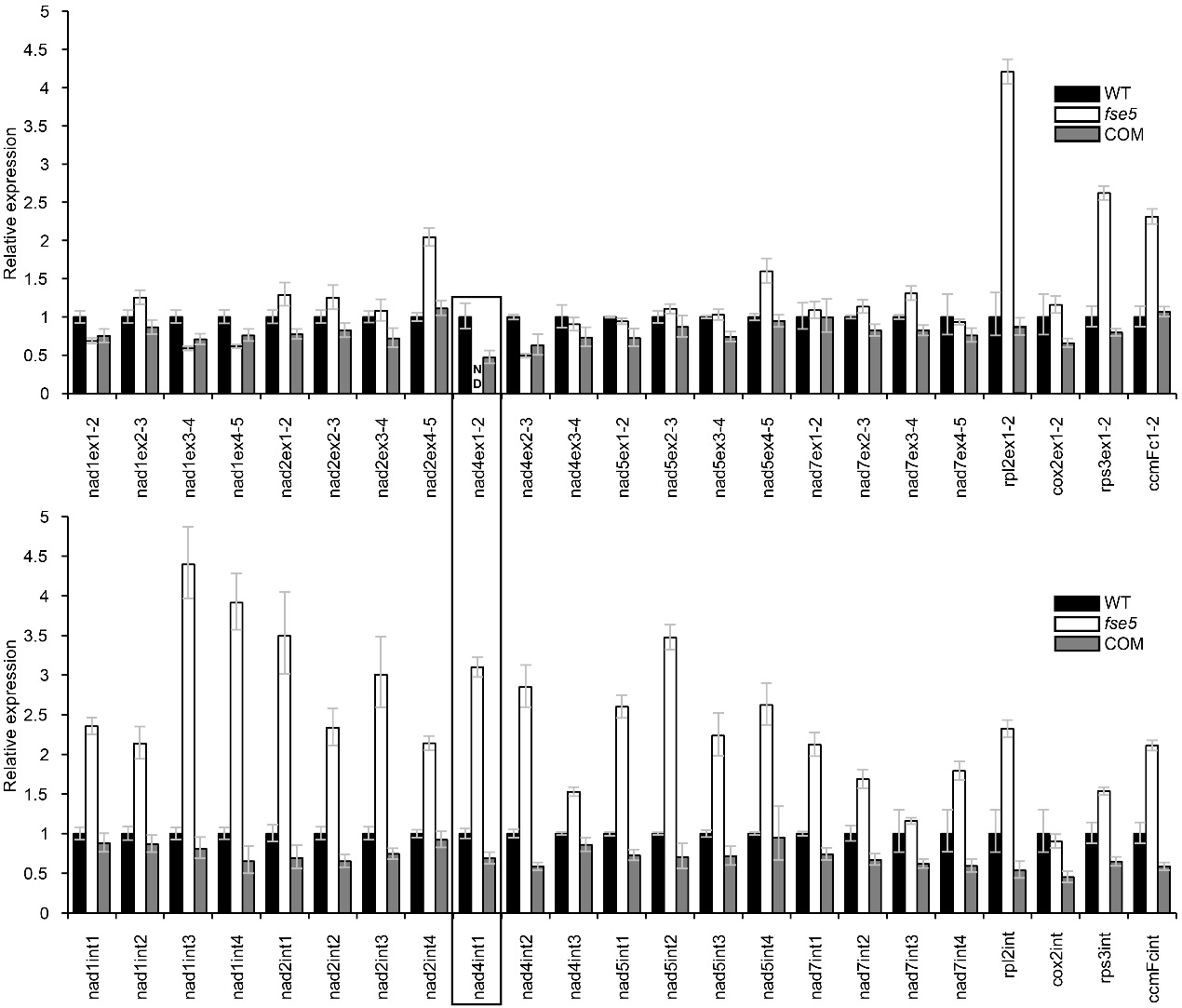


**Figure S5.** qRT-PCR analysis of 23 mitochondrial introns. Primers spanning adjacent exons were used to amplify fragments of mature mitochondrial transcripts (upper panel), and primers spanning adjacent exons and introns were used to amplify fragments of mitochondrial precursor mRNAs (lower panel) (Cai et al., 2017; Chen et al., 2017; Lee et al., 2017). The results of *nad4* exon 1-exon 2 (spliced fragment) and its precursor fragment are indicated by a black box. ND, not detected. Three biological replicates were assessed via qRT-PCR, and *OsActin1* was used as an internal control for data normalization. The values are the means ± SDs.


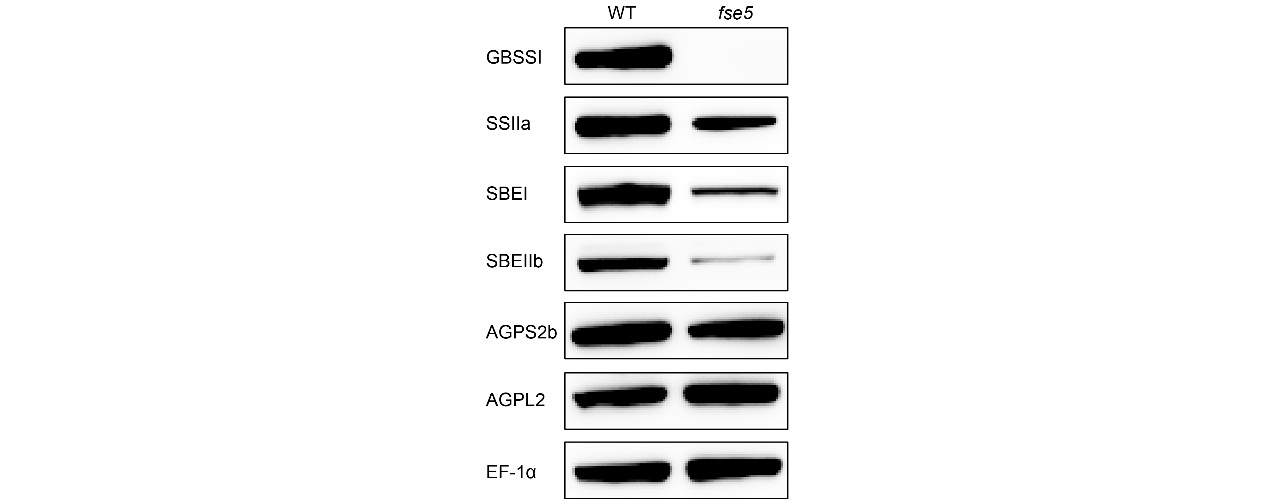


**Figure S6.** Immunoblot analysis of major starch synthases. Total proteins were extracted from developing WT and *fse5* seeds at 15 DAF and separated by SDS-PAGE (Takemoto et al., 2002; Wang et al., 2010). Translation elongation factor 1α (EF-1α), encoded by *Os03g0177400*, served as the loading control. GBSSI, Granule-bound starch synthase I. SSIIa, Starch synthase IIa. SBE, Starch branching enzyme. AGPS2b, ADP-glucose pyrophosphorylase 2b. AGPL2, ADP-glucose pyrophosphorylase large subunit 2.


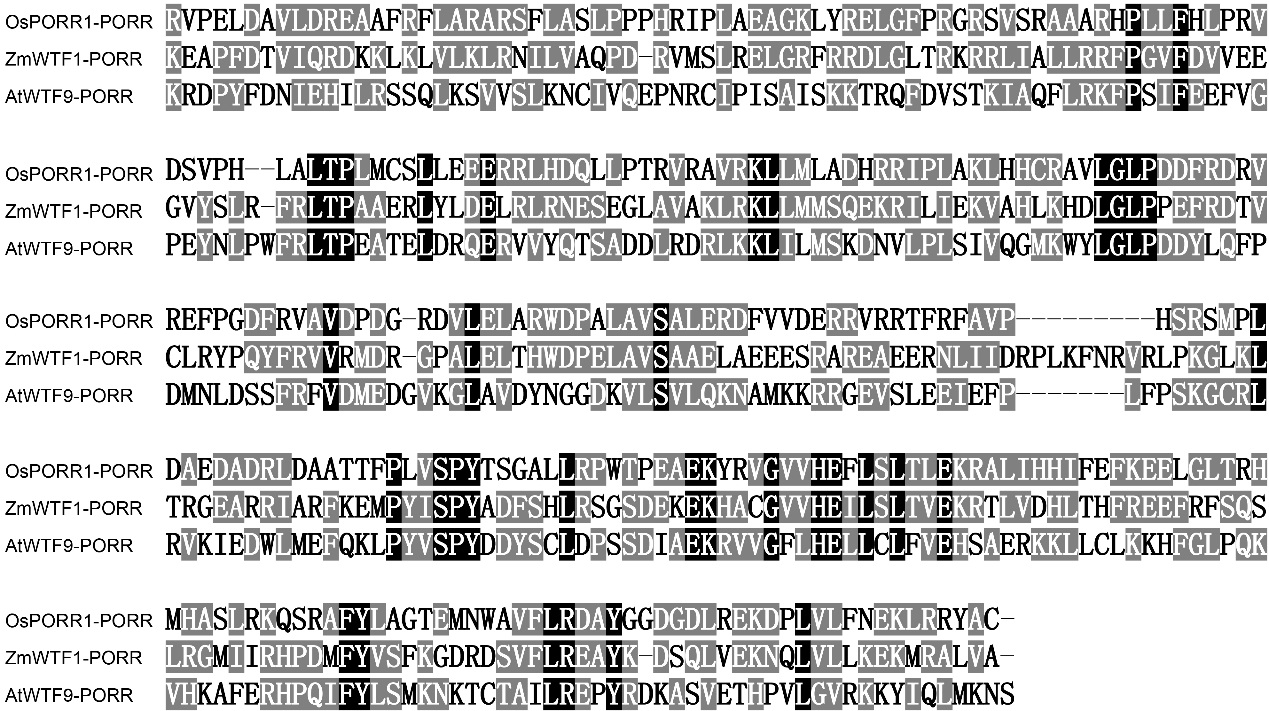


**Figure S7.** Multiple sequence alignment of PORR domains in OsPORR1, ZmWTF1 and AtWTF9. ZmWTF1, *GRMZM2G403797*; AtWTF9, *At2g39120*.


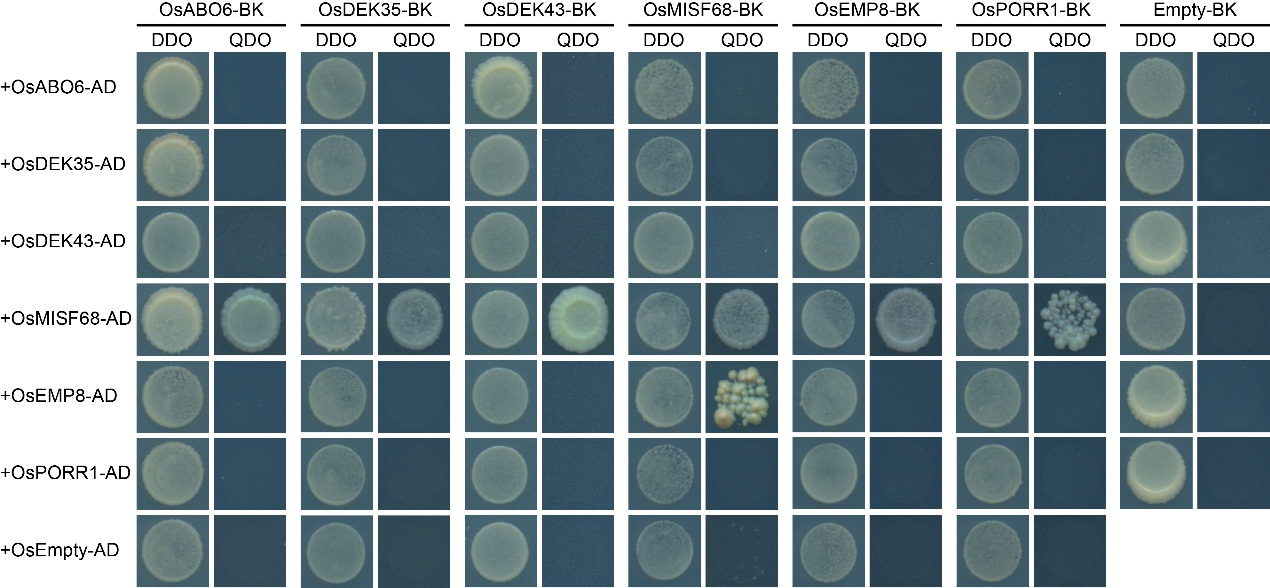


**Figure S8.** Y2H assays showing that MISF68 interacts together with splicing factors of *nad4* intron 1. Empty-AD and Empty-BK were used as controls. Full-length cDNAs of *OsABO6* (*LOC_Os01g02884*), *OsDEK35* (*LOC_Os03g50500*), *OsDEK43* (*LOC_Os05g11700*), *OsMISF68* (*LOC_Os02g16650*), *OsEMP8* (*LOC_Os08g41380*) and *OsPORR1* were cloned into pGADT7 or pGBKT7 vectors. The yeast transformation and screening procedures were performed according to the manufacturer’s instructions (TaKaRa Bio, Kusatsu, Japan). DDO, Double dropout media (SD/-Trp-Leu). QDO, Quadruple dropout media (SD/-Trp-Leu-His-Ade).

**Table S1.** Segregation of vitreous and floury grains from seven heterozygous plants (+/*fse5*)

| No. | Vitreous grains | Opaque grains | χ^2^_3:1*_ |
| --- | --- | --- | --- |
| 1 | 187 | 75 | 1.649 |
| 2 | 297 | 105 | 0.212 |
| 3 | 488 | 156 | 0.168 |
| 4 | 709 | 214 | 1.526 |
| 5 | 735 | 248 | 0.017 |
| 6 | 944 | 305 | 0.195 |
| 7 | 1003 | 336 | 0.002 |
| Total | 4363 | 1439 | 0.111 |

* The value for significance at *P* = 0.05 is 3.84.

**Table S2.** Primers used for mapping

| Primer name | Forward (5′→3′) | Reverse (5′→3′) |
| --- | --- | --- |
| RM242 | GGCCAACGTGTGTATGTCTC | TATATGCCAAGACGGATGGG |
| RM257 | CAGTTCCGAGCAAGAGTACTC | GGATCGGACGTGGCATATG |
| wi-9 | GAAACTTGAATAACGAGGAGG | GCAGTACGAGACCCGATT |
| wi-10 | CAAGTGGGATCTTTCCTGTC | TGCAGCGATTAACCGTGT |
| wi-11 | AATCATGTGCGATCTTCTCA | GAACAGAGTGCTGGTGGAGT |
| wi-19 | ATTTGGGAATATTCGGTGCT | TAACCAAGAGTTCCTGTCACG |
| wi-23 | TGTTGTGCCTTCTTGCC | ACCAGGAGCAATAATGCTT |
| wi-40 | TATGTGCCCGTAATGCTCT | ATACGGTGAAAGGTTGATCC |

**Table S3.** Primers used for vector construction

| Purpose | Primer name | Primer sequence (5’ to 3’) |
| --- | --- | --- |
| Complementation | 1390-PORR1-F | CCGGCGCGCCAAGCTTCCAAGTCCAGCAGATATTCAC |
|  | 1390-PORR1-R | GAATTCCCGGGGATCCCTAGTAGGCAGTAGCAATGCTC |
| KO | CRISPR-F | GGCACGCCGCTCATGTGCTCGCTCC |
|  | CRISPR-R | AAACGGAGCGAGCACATGAGCGGCG |
| Promoter analysis | 1381Z-PORR1-GUS-F | GAATTCCCGGGGATCCCCACCTGAGTTTCATTCTTATC |
|  | 1381Z-PORR1-GUS-R | GGCCAGTGCCAAGCTTCGAGTTCAGTCTATTTCGATCA |
| Subcellular localization | 1305-PORR1-GFP-F | CGGAGCTAGCTCTAGAATGGCGCGCCGCCTCTTC |
|  | 1305-PORR1-GFP-R | TGCTCACCATGGATCCGTAGGCAGTAGCAATGCTCTCA |
| Y2H (pGADT7) constructs | ABO6-AD-F | GGAGGCCAGTGAATTCATGCTCCTGGCCTCCTGCCTCCGG |
|  | ABO6-AD-R | CGAGCTCGATGGATCCTCAACCCCTCCTATGATGATGCCT |
|  | DEK35-AD-F | GGAGGCCAGTGAATTCATGTGGCGCGCGCCGATCCG |
|  | DEK35-AD-R | CGAGCTCGATGGATCCTCATCTAGGCTGCTGCCTTT |
|  | DEK43-AD-F | GGAGGCCAGTGAATTCATGAGCGGCGCCACCGCCAAGTTC |
|  | DEK43-AD-R | CGAGCTCGATGGATCCTCAAGCCAAGCTCAAATTATCCAG |
|  | EMP8-AD-F | GGAGGCCAGTGAATTCATGGGCCCAATCCTAACCTTACAC |
|  | EMP8-AD-R | CGAGCTCGATGGATCCTCAATTCCCATTCATCGACATCTC |
|  | EMP602-AD-F | GGAGGCCAGTGAATTCATGCCGCCCAAGCCAGAGCGGCGG |
|  | EMP602-AD-R | CGAGCTCGATGGATCCTCATCGTCGTGATTCAAATGTGTT |
|  | MISF68-AD-F | GGAGGCCAGTGAATTCATGGCGAGCCGCGCGCCGTGCCTC |
|  | MISF68-AD-R | CGAGCTCGATGGATCCTTATTCTTGATCAACCTTTCCAAT |
|  | PORR1-AD-F | GGAGGCCAGTGAATTCATGGCGCGCCGCCTCTTCTCCGCC |
|  | PORR1-AD-R | CGAGCTCGATGGATCCCTAGTAGGCAGTAGCAATGCTCTC |
| Y2H (pGBKT7) constructs | ABO6-BK-F | CATGGAGGCCGAATTCATGCTCCTGGCCTCCTGCCTCCGG |
|  | ABO6-BK-R | GCAGGTCGACGGATCCTCAACCCCTCCTATGATGATGCCT |
|  | DEK35-BK-F | CATGGAGGCCGAATTCATGTGGCGCGCGCCGATCCG |
|  | DEK35-BK-R | GCAGGTCGACGGATCCTCATCTAGGCTGCTGCCTTT |
|  | DEK43-BK-F | CATGGAGGCCGAATTCATGAGCGGCGCCACCGCCAAGTTC |
|  | DEK43-BK-R | GCAGGTCGACGGATCCTCAAGCCAAGCTCAAATTATCCAG |
|  | EMP8-BK-F | CATGGAGGCCGAATTCATGGGCCCAATCCTAACCTTACAC |
|  | EMP8-BK-R | GCAGGTCGACGGATCCTCAATTCCCATTCATCGACATCTC |
|  | EMP602-BK-F | CATGGAGGCCGAATTCATGCCGCCCAAGCCAGAGCGGCGG |
|  | EMP602-BK-R | GCAGGTCGACGGATCCTCATCGTCGTGATTCAAATGTGTT |
|  | MISF68-BK-F | CATGGAGGCCGAATTCATGGCGAGCCGCGCGCCGTGCCTC |
|  | MISF68-BK-R | GCAGGTCGACGGATCCTTATTCTTGATCAACCTTTCCAAT |
|  | PORR1-BK-F | CATGGAGGCCGAATTCATGGCGCGCCGCCTCTTCTCCGCC |
|  | PORR1-BK-R | GCAGGTCGACGGATCCCTAGTAGGCAGTAGCAATGCTCTC |

**Table S4.** Primers used for splicing analysis

| Primer name | Forward (5′→3′) | Reverse (5′→3′) |
| --- | --- | --- |
| *nad1*-RT | ATGTACATAGCTGTTCCAGCGGAAATA | TTAAGGGAGCCATCGAAAGGTGACTGA |
| *nad2*-RT | ATGTTCAATCTTTTTTTAGCGGTT | TTACAGATATGAACTGAGTGCCAT |
| *nad4*-RT | ATGTTAGAACATTTCTGTGAATGCTA | TCAATGAAATTTGCCATGTTGCACTA |
| *nad5*-RT | ATGTATCTACTTATTGTCTTTTTGCCT | TTATTCTTGACTTGACTTATTAATAA |
| *nad7*-RT | ATGACGACTAGGAACGGGCAAATCA | CTATCTATCTACCTCTCCAAACACA |
| *cox2*-RT | ATGATTCTTCGTTCATTAGAATGTCGA | TTAGTTGGTTTGGAGGATTAATTGA |
| *rpl2*-RT | ATGAGACAAAGCATAAAGGGGAGA | CACTCCTGCCCGAAATCCTGCTTT |
| *rps3*-RT | TTCGGTAAGACTTGATCTGAATCGTA | CTATATTTCGTACGTTTCGGATATA |
| *ccmFc*-RT | ATGGTCCAACTACAGAACTTCTTCT | ATTCCAATGCAACTTATCCTTTTGGA |
| *nad4*int-1 | TTGATTGGTCTGTGCGTTTC | GGCTTCGGGTAACCAAATA |
| *nad4*int-2 | TGCCGTCAAAGTGCCTAT | GGTGCTCACTAAACCTCCATA |
| *nad4*int-3 | CACTTTAGCCAATATGAGTTTACC | ATGCAATCCGGGAACACT |

**Table S5.** Primers used for qRT-PCR analysis

| Primer name | Forward (5′→3′) | Reverse (5′→3′) |
| --- | --- | --- |
| *Actin* | CCCTCCTGAAAGGAAGTACAGTGT | GTCCGAAGAATTAGAAGCATTTCC |
| *PORR1* | GAACTGGGCCGTGTTCCTCA | CCGCAGCTTCTCGTTGAACA |
| *AOX1a* | ACTTCGCATCGGACATCCATTA | AAATCCTCGGCAGTAGACAAAC |
| *AOX1b* | CGACCGACCCGCAGAACAAGA | CCCTTCGGCTCGTGGTGCTTC |
| *AOX1c* | ACACCGAGTACCTCAAGGACAT | AATGAACATCCGACGCGAAGTG |
| *NDA1* | ATAAGCATCTGGGGAGCATG | AAATACGGCTAATGTCACGG |
| *NDA2* | CTGCTCACCAACCTCATGCT | CTTGACGTAGTCCTTGACGT |
| *NDB1* | GAGTGGTAAAGGTCTCTGAT | GAAGCACAGTCACCAATTGC |
| *NDB2* | AAGGTACCCACAGGTTGAGT | GTTGAAGCAGTTTGCCAGAT |
| *NDB3* | AGCAGTTGGAGCTAGGTCTA | CAAAATCGTGCAACTCTGCG |
| *NDC1* | TGTGGGATTGGGTTACTCTG | GCTACTTGAATCCTCAGATG |
| nad1ex1-2 | TTGCCATATCTTCGCTAGGTG | GACCAATAGAGACTTCATAAGGGACCA |
| nad1int1 | CAAGTTGGGTTGGGGTATAGAG | TGAGCTGCAGATCGTAATGC |
| nad1ex2-3 | TCGAAATATGCCTTTCTAGGAG | ATTCAGCTTCCGCTTCTGG |
| nad1int2 | CTCGGATAAAAGCACGGACGAG | ATTCAGCTTCCGCTTCTGG |
| nad1ex3-4 | GTCATGGCGCAAAAGCAGATATGG | AGAGCAGACCCCATTGAAGA |
| nad1int3 | GTCGATTTATCCACACTTCCATGAC | AGAGCAGACCCCATTGAAGA |
| nad1ex4-5 | TGGGAGAGTATGCCAATATGA | ATCTAGGATAGGCGGCCAAC |
| nad1int4-1 | AGGAAAGCCCAACGAATGTCA | TCTTCAATGGGGTCTGCTC |
| nad2ex1-2 | TAGTTAGGAGAGGTGCGCCA | TGTGGGTTGGCTTGGATTACT |
| nad2int1 | CCCCATGCTAAGGTTCCCTG | TGTGGGTTGGCTTGGATTACT |
| nad2ex2-3 | AAAGGAACTGCAGTAATCTTGA | AATATTTGATCTTAGGTGCATTTCC |
| nad2int2 | GATCGAAGTGGGTAGCTCCA | GAGAGTCGCACGTACGGTAA |
| nad2ex3-4 | GCGCAATAGAAAGGAATGCT | CTATGGGTCTACTGGAGCTACCC |
| nad2int3 | CCCTCTCCCTCGGACTCAAT | AAAGGAATGCTGTCACCGGG |
| nad2ex4-5 | GTGGGGCTTACTTCCTAGCC | AGTAACGACTTGTCACGATCCA |
| nad2int4 | TCGGAGAGGACTCAGCTGTT | ACGACTTGTCACGATCCATTG |
| nad4ex1-2 | CCTGCATGCTGGATCCTCTA | ATCGGTGGTTCCTGTTTGGA |
| nad4int1 | CCTGCATGCTGGATCCTCTA | AAAGCTTGATCCCAGCGAAT |
| nad4ex2-3 | AATACCCATGTTTCCCGAAG | TGCTACCCCCAATTCCCTGT |
| nad4int2 | GTAACTATCTTGTACGGTTCGGA | CTAGTGCCGGGTAAACTCATATTG |
| nad4ex3-4 | GGCGTATTCCCTTTGGCTAT | CAATCCGGGAACACTTTGGG |
| nad4int3 | GAGACTATCTAGCTTGGTTCGGAG | CTTACGGATGTATGCATGCAATC |
| nad5ex1-2 | CATCGGAAATGTTTGATGCTTCTTG | AGAAAGTTATCTCCAGTCACCAACA |
| nad5int1 | CATCGGAAATGTTTGATGCTTCTTG | GATTCCCGACATGCTATGATACCC |
| nad5ex2-3 | TAATTCATTCGGGCGAGACAGATTA | TACCTAAACCAATCATCATATC |
| nad5int2 | TAATTCATTCGGGCGAGACAGATTA | ATGATCGTGTTGGGTAAATTGTGAC |
| **Continued Table S5** | | |
| nad5ex3-4 | GATATGATGATTGGTTTAGGTA | AACTCGGATTCGGCAAGAA |
| nad5int3 | ATATGATGATTGGTTTAGGTA | TATGTTTCCCTTCTTCCATTCTCAA |
| nad5ex4-5 | GTCAGATCGTTCCTGCGTTT | GGTCACAAATGGAGTTGAACCA |
| nad5int4 | CACCAACCAAAGCAGGTAGG | TCTCGTACACATTCCGACGA |
| nad7ex1-2 | TGCTGCTCATGGTGTTTCAC | CTCGATTAATTTCTCAGTCCCTC |
| nad7int1 | TGAACGGAGAAGTGGTGGAA | CATAGGAGCCTACTCAGCGT |
| nad7ex2-3 | GAGGGACTGAGAAATTAATCGAG | TGGTACCTCACAATTCAAAA |
| nad7int2 | ACGGTTCAGAGAGCACTTGT | GGCTGAAGAATGAGCGTGTT |
| nad7ex3-4 | TGCACAGCAAGCAAAGGATT | TCCGAACACTTTGTCGCATC |
| nad7int3 | GCCTCTTGGCTTATGTCGAG | ATATGCATGCTTTTGTAGGGTCA |
| nad7ex4-5 | AGATGCGACAAAGTGTTCGG | AAGGAGCTGGTACGGAAAAAC |
| nad7int4 | AGATGCGACAAAGTGTTCGG | ATGCTTTACTCCTAACCCCACG |
| cox2ex1-2 | GCTCTGTTATACTCAATGGACGGG | AGATGAGTTTTGGCTGGTACAACC |
| cox2int | GCTCTGTTATACTCAATGGACGGG | GAGCATTTCGGGGTATAGGTCTAA |
| ccmFcex1-2 | TCACATGGAGGAGTGTGCAT | CGAGACCTCGCAAACAACAA |
| ccmFcint | TTGAAGCTCTCGCCTTACCA | CAGGTCGCACATAAGCCATC |
| rpl2ex1-2 | GGATGGAGCGTACAAAGTCG | GAAGGTTTGGAGCAATCGCA |
| rpl2int | GGATGGAGCGTACAAAGTCG | TGTTTCTAGGTGGGTCGCTT |
| rps3ex1-2 | CCAATTTCGGTAAGACTTGA | AGCCAAAGGTGAGTATCGTA |
| rps3int | TTTCGGTAAGACTTGATCTGAATCG | GCGTTAGAAGAAGTCGTGTCC |

**References**

Cai M, Li S, Sun F, Sun Q, Zhao H, Ren X, Zhao Y, Tan B, Zhang Z, Qiu F (2017) *Emp10* encodes a mitochondrial PPR protein that affects the *cis*-splicing of *nad2* intron 1 and seed development in maize. Plant J 91:132-144

Chen X, Feng F, Qi W, Xu L, Yao D, Wang Q, Song R (2017) *Dek35* encodes a PPR protein that affects *cis*-splicing of mitochondrial *nad4* intron 1 and seed development in maize. Mol Plant 10:427-441

Lee K, Han JH, Park YI, Colas des Francs-Small C, Small I, Kang H (2017) [The mitochondrial pentatricopeptide repeat protein PPR19 is involved in the stabilization of NADH dehydrogenase 1 transcripts and is crucial for mitochondrial function and *Arabidopsis thaliana* development.](https://www.ncbi.nlm.nih.gov/pubmed/28332713) New Phytol 215:202-216

[Takemoto Y](https://www.ncbi.nlm.nih.gov/pubmed/?term=Takemoto%20Y%5bAuthor%5d&cauthor=true&cauthor_uid=11950970), [Coughlan SJ](https://www.ncbi.nlm.nih.gov/pubmed/?term=Coughlan%20SJ%5bAuthor%5d&cauthor=true&cauthor_uid=11950970), [Okita TW](https://www.ncbi.nlm.nih.gov/pubmed/?term=Okita%20TW%5bAuthor%5d&cauthor=true&cauthor_uid=11950970), [Satoh H](https://www.ncbi.nlm.nih.gov/pubmed/?term=Satoh%20H%5bAuthor%5d&cauthor=true&cauthor_uid=11950970), [Ogawa M](https://www.ncbi.nlm.nih.gov/pubmed/?term=Ogawa%20M%5bAuthor%5d&cauthor=true&cauthor_uid=11950970), [Kumamaru T](https://www.ncbi.nlm.nih.gov/pubmed/?term=Kumamaru%20T%5bAuthor%5d&cauthor=true&cauthor_uid=11950970) (2002) The rice mutant *esp2* greatly accumulates the glutelin precursor and deletes the protein disulfide isomerase. Plant Physiol 128:1212-1222

Wang Y, Ren Y, Liu X, Jiang L, Chen L, Han X, Jin M, Liu S, Liu F, Lv J, Zhou K, Su N, Bao Y and Wan J (2010) OsRab5a regulates endomembrane organization and storage protein trafficking in rice endosperm cells. Plant J 64:812-824

Zhang C, Muench DG (2015) A nucleolar PUF RNA-binding protein with specifcity for a unique RNA sequence. J Biol Chem 290:30108-30118
